# No phylogenomic support for a Cenozoic origin of the “living fossil” *Isoetes*

**DOI:** 10.1002/ajb2.16108

**Published:** 2023-01-02

**Authors:** Niklas Wikström, Eva Larsén, Anbar Khodabandeh, Catarina Rydin

**Affiliations:** ^1^ Bergius Foundation, The Royal Swedish Academy of Sciences Box 50005 SE‐104 05 Stockholm Sweden; ^2^ Department of Ecology, Environment, and Plant Sciences Stockholm University SE‐106 91 Stockholm Sweden

**Keywords:** Bayesian inference, clock model, independent lognormal model (ILN), Isoetaceae, Isoetales, lycopods, Mesozoic, node ages, Thorne and Kishino model (TK02), white noise model (WN)

## Abstract

**Premise:**

The isoetalean lineage has a rich fossil record that extends to the Devonian, but the age of the living clade is unclear. Recent results indicate that it is young, from the Cenozoic, whereas earlier work based on less data from a denser taxon sampling yielded Mesozoic median ages.

**Methods:**

We analyzed node ages in *Isoetes* using two genomic data sets (plastome and nuclear ribosomal cistron), three clock models implemented in MrBayes (ILN, WN, and TK02 models), and a conservative approach to calibration.

**Results:**

While topological results were consistently resolved in *Isoetes* estimated crown group ages range from the latest Paleozoic (mid‐Permian) to the Mesozoic depending on data type and clock model. The oldest estimates were retrieved using the autocorrelated TK02 clock model. An (early) Cenozoic age was only obtained under one specific condition (plastome data analyzed with the uncorrelated ILN clock model). That same plastome data set also yielded the oldest (mid‐Permian) age estimate when analyzed with the autocorrelated TK02 clock model. Adding the highly divergent, recently established sister species *Isoetes wormaldii* to the data set approximately doubled the average median node depth to the *Isoetes* crown group.

**Conclusions:**

There is no consistent support for a Cenozoic origin of the living clade *Isoetes*. We obtained seemingly well‐founded, yet strongly deviating results depending on data type and clock model. The single most important future improvement is probably to add calibration points, which requires an improved understanding of the isoetalean fossil record or alternative bases for calibration.

Lycopods (Lycopodiophyta) are an ancient group of plants with an extensive fossil record dating back at least to the Early Devonian, some 400 million years ago (Ma) (Bateman and DiMichele, 1994; Kenrick and Crane, 1997a; Edwards and Kenrick, 2015; Morris et al., 2018). The group comprises three extant lineages: the homosporous Lycopodiaceae and the two ligulate and heterosporous lineages Selaginellaceae and Isoetaceae. The fossil record clearly indicates that these three extant lineages had already diverged in the Paleozoic. *Leclercqia complexa* Banks (Banks et al., 1972), a homosporous but ligulate plant, has been documented from the Emsian (late Early Devonian) of Canada (Gensel and Albright, 2006; Wellman et al., 2009) and indicates a minimum of 392 Myr since the split between the Lycopodiaceae and a ligulate clade comprising *Leclercqia* and the extant lineages Isoetaceae and Selaginellaceae (Kenrick and Crane, 1997a; Wellman et al., 2009; Morris et al., 2018). Isoetalean arborescent fossils, including their “rhizomorph” shoot structure producing stigmarian rootlets, are known at least from the Famennian (late Late Devonian) (Pigg, 2001; Wang and Berry, 2003; Cressler and Pfefferkorn, 2005; Taylor et al., 2009; Prestianni and Gess, 2014; Hetherington and Dolan, 2017; Gerrienne et al., 2018; DiMichele et al., 2022). The fossils indicate a minimum of 358 Myr since the split between extant Isoetaceae and Selaginellaceae (Bateman, 1992; Bateman and DiMichele, 1994; DiMichele and Bateman, 1996; Kenrick and Crane, 1997a; Hetherington and Dolan, 2017).

Less is known about the origins and early diversification of the extant lycopod crown groups. Although the fossil record includes taxa with considerable similarities to all three extant lineages (Pigg, 1992; Skog and Hill, 1992; Thomas, 1992; Retallack, 1997; Thomas, 1997; Pigg, 2001; Taylor et al., 2009; McLoughlin et al., 2014; Schmidt et al., 2020), their specific relationships to extant species are often unclear. Selaginellaceae are probably the least problematic group. Here, a well‐supported phylogenetic framework has been developed over the last 20 years (Korall et al., 1999; Korall and Kenrick, 2002, 2004; Arrigo et al., 2013; Weststrand and Korall, 2016; Zhou et al., 2016; Zhang et al., 2020), and ingroup morphological synapomorphies, such as flattened shoots, anisophyllous leaves, and presence of rhizophores, have been possible to identify also in fossils. Fossil taxa such as *Selaginella labutae* Bek, Libertín & Drábková (Bek et al., 2009) from the Moscovian (late Carboniferous), and *S. anasazia* Ash (Ash, 1972) from the Late Triassic, have confidently been placed as part of the ingroup *Selaginella* (Korall and Kenrick, 2002; Bek et al., 2009). These indicate a minimum age for the *Selaginella* crown group of ca. 300 Myr. A corresponding phylogenetic framework has been developed also in the homosporous Lycopodiaceae (Wikström and Kenrick, 1997; Wikström et al., 1999; Wikström and Kenrick, 2000; Wikström, 2001; Ji et al., 2008; Burnard et al., 2016; Field et al., 2016; Testo et al., 2018; Chen et al., 2022), but here ingroup morphological synapomorphies are less clear. Wikström (2001) tentatively placed *Lycoxylon indicum* Srivastava (Srivastava, 1946), *Lycopodites falcatus* Lindley & Hutton (Lindley and Hutton, 1833), and specimens of the fossil spore taxon *Retitriletes* Pierce (Pierce, 1961) as ingroup members of the Lycopodiaceae crown group. *Lycoxylon indicum* has a plectostele, similar to the type seen in *Lycopodium* s.l. (Srivastava, 1946; Wikström, 2001), *Lycopodites falcatus* is vegetatively reminiscent of *Lycopodium* section *Complanata* (Harris, 1961; Skog and Hill, 1992), and the spore genus *Retitriletes* includes specimens highly similar to the reticulate spores found in *Lycopodium* s.l. (Wilce, 1972; Dettmann, 1986). When considered together, they indicate a minimum age for the Lycopodiaceae crown group of ca. 200 Myr (Wikström, 2001). More recently, Herrera et al. (2022) documented a crown‐group Lycopodiaceae fossil from a chert deposit in Mongolia of Barremian to earliest Aptian age (126 Myr). Using a combined DNA and morphological data matrix they placed the fossil within the subfamily Lycopodioideae (*Lycopodium* s.l.), firmly establishing the Lycopodiaceae crown group in the mid Early Cretaceous (Herrera et al., 2022).

Isoetaceae have proven the most problematic group. A single genus (*Isoetes* L.) is most often recognized, comprising no more than 200–250 species, and with a more or less worldwide distribution (PPG I, 2016; Troìa et al., 2016). Most species are semi‐aquatic (or amphibious) herbs, but fully aquatic and strictly terrestrial species are also found (Pfeiffer, 1922; Jermy, 1990; Troìa et al., 2016). Early attempts at recognizing subgeneric groups in *Isoetes* put much emphasis on these different habits (Baker, 1880; Engelmann, 1882; Motelay and Vendryès, 1882), but transitions between aquatic, semiaquatic, and terrestrial habits have been inferred to have occurred multiple times within different lineages of the genus (Hickey, 1986; Taylor and Hickey, 1992). More recent morphology‐based works have emphasized other characters such as megaspore morphology and/or features of the leaves (Pfeiffer, 1922; Fuchs‐Eckert, 1982; Hickey, 1986, 1990; Taylor and Hickey, 1992), but morphological stasis coupled with ample variation and parallel evolution within that conserved framework have made it problematic to establish any ingroup morphological synapomorphies (Freund et al., 2018). As an example, the set and subset of leaf characters hypothesized to be characteristic of early‐diverging species of *Isoetes* by Hickey (1986) were not unambiguously supported as the plesiomorphic condition in subsequent molecular‐based analyses (Rydin and Wikström, 2002; Larsén and Rydin, 2016). Also, megaspore structure (Pfeiffer, 1922) appears inadequate for identifying any clear subgroups of *Isoetes* (Cox and Hickey, 1984; Larsén and Rydin, 2016). Consequently *Isoetes*‐like fossil plants described from the Mesozoic and the Cenozoic (see e.g., Pigg, 1992, 2001; Skog and Hill, 1992 for reviews) cannot be unequivocally placed within the crown group of extant species (Pigg, 2001), and our fossil‐based age estimate for the origin and diversification of extant *Isoetes* is tentative at best.

Molecular systematics has addressed this issue in a different way. Using primarily plastid and nuclear ribosomal DNA (rDNA) data, phylogenetic analyses have produced a coherent and well‐supported phylogenetic framework of *Isoetes* (Rydin and Wikström, 2002; Hoot et al., 2004, 2006; Schuettpelz and Hoot, 2006; Larsén and Rydin, 2016; Pereira et al., 2017; Larsén et al., 2022). Five major clades within *Isoetes* have been supported: a Gondwanan (Clade A), a Laurasian (Clade B), an Italian‐Turkish (Clade C), an Austro‐Asian (Clade D), and a New World or American clade (Clade E). These molecular‐based analyses have dramatically changed our understanding of phylogenetic relationships in *Isoetes* but finding morphological features supporting any one of the inferred subgroups remain a challenge (Larsén and Rydin, 2016; Freund et al., 2018). A recent analysis by Larsén et al. (2022, p. 1) expanded on this theme further by reporting an “*Amborella* syndrome”, a divergent and poorly known species (*Isoetes wormaldii*) being sister to the rest of the family, and by adding to the geographical distributions included in the five subgroups.

Following phylogenetic analyses, the temporal origin and diversification of *Isoetes* have subsequently been addressed using relaxed‐clock methods (Kim and Choi, 2016; Larsén and Rydin, 2016; Wood et al., 2020; Pereira et al., 2017, 2021). However, these studies have shown highly divergent results. Larsén and Rydin (2016) used plastid (*rbcL*, *rbcL‐atpB* spacer) and nuclear (nrITS spacer) data and a broad taxon sampling that covered all major groups of land plants and estimated the median age of crown group *Isoetes* as latest Jurassic (147; 96–215 Myr). Two circumstances were seen by the authors as consistent with this result: the match between the estimated age of the crown group and the age of the oldest known fossil with an *Isoetes* habit, *Isoetites rolandii* from the Late Jurassic of Idaho (Ash and Pigg, 1991; Pigg, 2001) and the diverse geographical distribution of the species included in several of their inferred clades (Clades A–E; Larsén and Rydin, 2016). These distributions were partly interpreted as related to the initial breakup of the Gondwana continent during the Mesozoic (Seton et al., 2012). In stark contrast to these results that indicate a Mesozoic age of the crown group *Isoetes*, Wood et al. (2020) inferred *Isoetes* to have diversified in the Cenozoic. Using a plastid genome data set and a data set of nuclear orthologs, they inferred the median age of crown group *Isoetes* as either Oligocene–Miocene boundary (23; 6–47 Myr; plastid genome data) or early Eocene (54; 28–85 Myr; nuclear ortholog data). Compared with the analyses by Larsén and Rydin (2016), they included a somewhat meager sample of *Isoetes* species (7 compared with 44) and fewer representatives of other lycopods (3 compared with 35), although they did include representatives of all the major land plant lineages. Their strongly deviating result compared with previous work with a relatively recent diversification of extant *Isoetes* (Wood et al., 2020) was surprising, but subsequent analyses by Pereira et al. (2021) also indicated a very recent diversification of extant *Isoetes* in the early Miocene (20; 15–26 Ma). A comparison of their results with those of Larsén and Rydin (2016) and Wood et al. (2020) is complicated by different outgroup sampling; Pereira et al. (2021) did not include any representatives of other major land plant lineages than lycopods. Their results have, however, contributed to the view that modern day *Isoetes* may be a much younger group than previously thought (Wood et al., 2020; Pereira et al., 2021).

The present study attempts to evaluate these different age estimates of the crown group *Isoetes* using a phylogenomic approach and methodological comparison. Using data from complete plastid genomes and the nuclear ribosomal cistron, we conducted a series of non‐clock and relaxed‐clock analyses. The taxon sampling from Wood et al. (2020) was used as a starting point but expanded considerably, particularly with respect to the number of included species of *Isoetes*. In contrast to previous studies, we also evaluated how alternative relaxed‐clock models impact our inferred node ages.

## MATERIALS AND METHODS

### Taxon sampling

We selected 25 ingroup (*Isoetes*) samples representing 24 different species for the present study. The selection was done with the aim to broadly cover the geographic distribution and the phylogenetic diversity of *Isoetes* as indicated by previous work (Hoot et al., 2006; Larsén and Rydin, 2016; Pereira et al., 2017; Larsén et al., 2022). One previously sequenced plastome (from *Isoetes flaccida* Karol et al., 2010) was included in the plastid data set. In addition, 25 outgroup terminals were included to represent major land plant groups. Outgroups were selected to use a taxon sampling corresponding to that used in the analyses by Wood et al. (2020). Taxon names, voucher information, geographic distributions of included *Isoetes* species, and collection localities of the voucher specimens are given in Appendix S1.

### DNA extraction and sequencing

Total genomic DNA was extracted from the selected specimens using the cetyltrimethylammonium bromide (CTAB) protocol (Doyle and Doyle, 1987; Doyle, 1991). Extractions were cleaned using the QIAquick PCR cleaning kit (Qiagen, Hilden, Germany). High‐throughput sequencing was performed at the Science for Life Laboratory (SciLifeLab, Stockholm, Sweden) following the manufacturer's instructions for the Illumina HiSeq. 2500 platform (Illumina, San Diego, CA, USA). Pair‐end runs with 350‐bp insert size fragments and 2 × 125 bp read lengths were performed. Samples were multiplexed with a total of 93 samples and run in three lanes. Library preparation at the SciLifeLab was done using the ThruPLEX DNA‐seq library preparation kit from Rubicon (Rubicon Genomics, Ann Arbor, MI, USA). Demultiplexing and conversion was conducted using bcl2fastq v.2.17 from the CASAVA software suite (Illumina). Single raw reads from the Illumina sequencing were set in pairs, merged using the BBmerge function, and low‐quality nucleotides were removed with the error probability limit 0.05 in Geneious v.10.2.6 (https://www.geneious.com).

### Plastid sequence assembly

To assemble the reads, a reference‐guided genome skimming approach was used (Straub et al., 2012). Plastid sequences were isolated from the original reads using a BLAT (BLAST‐like alignment tool v36, Kent, 2002) search of forward and reverse reads against a reference database. The initial database comprised the complete plastid genome of *Isoetes flaccida* (NC_014675, Karol et al., 2010), but assembled plastids were successively added to the database once completed. Forward and reverse reads were extracted if either showed at least 70% similarity to any of the reference genomes. Following the BLAT search, reads were extracted from the original fastq data files using pullseq v.1.0.1 (github.com/bcthomas/pullseq) into new forward and reverse “plastid” data files. De novo assembly of this “plastid” subset of reads was performed for each taxon using ABySS v.2.3.4 (Simpson et al., 2009; Jackman et al., 2017) and seven *k*‐mer lengths (55, 61, 67, 73, 85, 91, 97). Generated contigs were pooled and mapped onto a reference genome using bwa v.0.7.17‐r1188 (Li and Durbin, 2009). Initially, the plastid genome of *Isoetes flaccida* (NC_014675) was used as reference, resulting in complete or nearly complete draft genomes. All original reads were subsequently mapped onto the draft genomes using bwa v.0.7.17‐r1188 allowing unfinished gaps to be filled and sequencing depths evaluated. Generated assemblies were reviewed and edited using gap5 from the Staden Package (Staden, 1996; Staden et al., 2000; Bonfield and Whitwham, 2010). Once a plastid genome was assembled, it was added to the database of plastid genomes used in the BLAT search and also considered as a potential reference genome in the final mapping stage in subsequent assemblies.

### Assembly of nuclear rDNA cistrons

Nuclear ribosomal DNA (rDNA) cistrons were assembled in a way similar to that used for the plastids. An initial reference sequence including partial external transcribed spacer (ETS), 18S gene, internal transcribed spacer 1 (ITS1), 5.8S gene, internal transcribed spacer 2 (ITS2), and 26S gene sequences was constructed based on the *Isoetes* sp. (PYHZ) transcriptome from the One Thousand Plant Transcriptomes Initiative (2019). The transcriptome sequence was aligned against a complete 18S sequence from *Isoetes durieui* (Kranz and Huss, 1996); ITS1, 5.8S, and ITS2 sequences from *Isoetes olympica* (Bolin et al., 2011); partial 26S sequence from *Selaginella selaginoides* (Korall and Kenrick, 2004); and an rDNA cistron sequence of *Asclepias syriaca* (Straub et al., 2011).

### Phylogenetic analyses

#### Plastid data

Protein‐coding sequences (CDS) were extracted from the annotated GenBank files using a Python script developed in‐house and made available at the Dryad Digital Repository (https://doi.org/10.5061/dryad.q573n5tf4; Wikström et al., 2020). Stop codons and regions of uncertain alignment were identified by eye and removed using a Python script developed by Fučíková et al. (2016) and made available at the Dryad Digital Repository (https://doi.org/10.5061/dryad.q8n0v). Outgroup sequence files were downloaded from GenBank (Appendix S1). All regions were aligned individually using MUSCLE v.3.8.31 (Edgar, 2004) as amino‐acid sequences and converted back to nucleotides using Seaview 4 (Gouy et al., 2009). Regions absent in significant numbers of taxa, or highly divergent and therefore difficult to confidently align across all taxa were not included in the final data set. Altogether 66 CDS regions were included in the final data set with a total of 50 taxa and 47,547 nucleotide characters. Data sets are available in the Dryad Digital Repository (https://doi.org/10.5061/dryad.q2bvq83pc; Wikström et al., 2022).

##### Non‐clock analyses

Protein‐coding genes were concatenated into a combined CDS data set. Bayesian analyses were conducted using a development version of MrBayes v.3.2.7a (Huelsenbeck and Ronquist, 2001; Ronquist and Huelsenbeck, 2003). The CDS data were partitioned by codon position. The substitution model for each partition was chosen based on the corrected Akaike information criterion (AICc) as calculated using MrAIC v.1.4.6 (Nylander, 2004) and PhyML v.3.0 (Guindon and Gascuel, 2003; Guindon et al., 2010). The GTR + I + G substitution model was chosen for all three partitions. Four independent runs were conducted, each including 10,000,000 generations, and stationarity of each chain was assessed using Tracer v.1.7 (Rambaut et al., 2018). Following stationarity, trees and parameters were sampled every 1000 generations in each chain. Convergence of the chains was assessed by comparing their posterior distributions, and trees and parameter samples from each chain were subsequently pooled to produce a final posterior distribution of 20,000 samples.

##### Relaxed‐clock analyses

Relaxed‐clock analyses were conducted using a development version of MrBayes v.3.2.7a. As in the non‐clock analyses the CDS data were partitioned by codon position. Three alternative relaxed‐clock models were used, the independent lognormal model (ILN, Drummond et al., 2006), the white noise model (WN, LePage et al., 2007), and the Brownian motion model described by Thorne and Kishino (TK02, Thorne and Kishino, 2002). MrBayes runs were conducted similar to those in the non‐clock analyses. Two fossil calibrations were used in the analyses, one for the crown group land plants, and one for the split between extant Selaginellaceae and Isoetaceae. A uniform prior age distribution with a maximum age of 485 Myr and a minimum age of 421 Myr was applied to the age of land plants (our root node). Using the Geologic Time Scale v.5.0 (Walker et al., 2018), we based the lower (older) limit on the appearance of cryptospores in nonmarine fossil deposits during the Middle Ordovician (Strother et al., 1996; Rubinstein et al., 2010; Wellman, 2010), spores that likely represent our earliest available evidence for the occurrence of plants on land (Gray, 1985; Kenrick and Crane, 1997a, b; Wellman et al., 2003; Gensel, 2008; Morris et al., 2018). While distinct (and adapted for survival and dispersal), such spores have not unequivocally been documented from pre‐Ordovician deposits (Kenrick, 2003; Wellman et al., 2003; Wellman, 2010), and we therefore used the Cambrian–Ordovician boundary at 485 Ma to set the maximum age for our land plant node. The upper (younger) age limit for our land plant node is based on *Zosterophyllum* sp. (Kotyk et al., 2002) and *Baragwanathia longifolia* (Lang and Cookson, 1935), both documented from the late Silurian (Ludlow). They are both part of the total group tracheophytes and provide unequivocal minimum age estimates for the origin of land plants (Kenrick and Crane, 1997a; Morris et al., 2018). Following the approach of Morris et al. (2018), we used a minimum age of 421 Myr based on these fossils. Our second calibration, the split between extant Selaginellaceae and Isoetaceae, was constrained using a uniform prior age distribution with a maximum age of 485 Myr (same as our root node) and a minimum age of 358 Myr. The minimum age is based on multiple occurrences of isoetalean arborescent lycopsids in deposits of Late Devonian (Famennian) age (Pigg, 2001; Wang and Berry, 2003; Cressler and Pfefferkorn, 2005; Prestianni and Gess, 2014; Gerrienne et al., 2018). Morris et al. (2018) treated the fossil taxa considered here less conservatively than we have. On the basis of similarities to extant liverworts (Brown et al., 2015; Renzaglia et al., 2015), they placed the Middle Ordovician cryptospores as part of the crown group of extant land plants and used a minimum age constraint of 469 Myr for their crown group Embryophyta (Morris et al., 2018). Furthermore, they considered *Zosterophyllum* a member of their Lycopodiophyta total group and used 421 Ma as a minimum for the divergence of tracheophytes into their Lycopodiophyta and Euphyllophyta (Morris et al., 2018). We have not followed Morris et al. (2018) regarding their choice of calibration to absolute time, but have prioritized using the same fossil‐based age constraints as those used by Wood et al. (2020). However, if treated as done by Morris et al. (2018), time estimates of early divergences would be earlier than those resulting from our analyses.

#### Nuclear rDNA data

Nuclear 18S, 5.8S, and 26S rDNA gene regions were extracted from the annotated GenBank files using the same in‐house Python script used for plastid sequences. Outgroup sequences were extracted from transcriptomes produced by the 1000 plant (1KP) transcriptome initiative (One Thousand Plant Transcriptomes Initiative, 2019). Transcriptomes used were selected to mirror, as closely as possible, the taxon sampling used in the plastid‐based analyses (see Appendix S1). Each region was aligned individually using MUSCLE v.3.8.31 (Edgar, 2004). Altogether, the final rDNA data set comprised 49 taxa and 5665 nucleotide characters. Data sets were deposited in the Dryad Digital Repository (https://doi.org/10.5061/dryad.q2bvq83pc; Wikström, et al., 2022).

##### Non‐clock analyses

Non‐clock analyses were conducted on a concatenated data set, and data were partitioned into a single partition. The substitution model was chosen based on the AICc, as calculated using MrAIC v.1.4.6 (Nylander, 2004) and PhyML v.3.0 (Guindon and Gascuel, 2003; Guindon et al., 2010), resulting in the GTR + I + G model. Analyses were in all other aspects set up as done for the plastid‐based analyses.

##### Relaxed‐clock analyses

Relaxed‐clock analyses were also conducted on the concatenated data set. As in the plastid‐based analyses, three alternative relaxed‐clock models were used (ILN, WN, and TK02), and all aspects of the analyses were set up as done for the plastid‐based analyses.

## RESULTS

### Assembled plastids

Complete plastid sequences were successfully assembled for 23 *Isoetes* accessions. Assembled sequences ranged in length from 143,143 bp in *Isoetes cubana* to 145,411 bp in *I. neoguineensis*. The total number of sequenced fragments varied across samples from 6.3 × 10^6^ fragments in *Isoetes weberi* to 18.3 × 10^6^ fragments in *I. neoguineensis*, yielding an average sequencing depth for the plastids around 60 reads. Assembled sequences were submitted to the GenBank database, and information on all deposited sequences is given in Appendix S1. *Isoetes philippinensis* (EL036) had poor sequencing quality and shallow sequencing depth, and a complete plastid sequence was not assembled for this taxon. Here, the 66 CDS regions included in the phylogenetic analyses were assembled and submitted as separate sequences.

### Assembled rDNA cistrons

Nuclear rDNA cistrons were assembled for the corresponding accessions. Assembled sequences ranged in length from 6445 bp in *I. wormaldii* to 7035 bp in *I. malinverniana* and comprised partial external transcribed spacer (ETS), 18 S, ITS1, 5.8 S, ITS2, and 26 S ribosomal DNA sequence. Average sequencing depths for the rDNA cistrons varied between ca. 300 reads (*I. philippinensis*) to more than 2500 reads (*I. lechleri*). See Appendix S1 for a comprehensive list of accession numbers for all deposited sequences.

### Phylogenetic analyses

#### Non‐clock analyses

**Figure 1:**
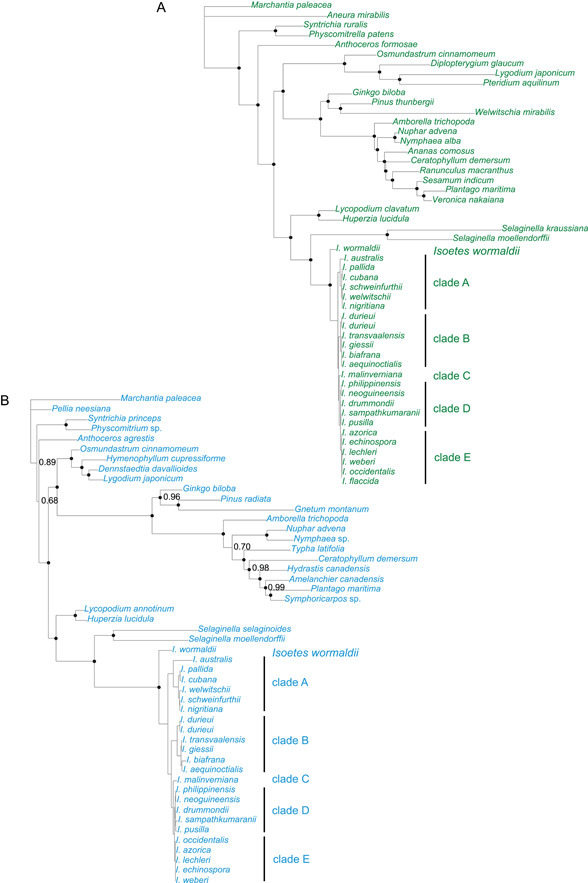
Phylograms resulting from non‐clock analyses of (A) complete plastid CDS data and (B) nuclear rDNA gene data (18S, 5.8S, 26S). *Isoetes wormaldii* and the clades A–E are discussed in the text. Nodes indicated by a black dot are well supported and have a Bayesian posterior probability (BPP) of 0.95 or more (Alfaro et al., 2003; Erixon et al., 2003). The BPP values are 1.00 unless otherwise indicated in the figure. Expanded versions of the phylograms also showing branch support for internal branches in *Isoetes* are reported in Appendix S2, Figures S1 and S2.

Phylograms resulting from the non‐clock analyses of complete plastid CDS data and of data from the nuclear rDNA genes (18S, 5.8S, and 26S) are reported in Figure 1 (A: plastid data; B: nuclear rDNA data). Expanded versions of the phylograms also showing branch support for internal branches in *Isoetes* are reported in Appendix S2 (Figures S1 and S2).

Relationships in *Isoetes* are with a few exceptions well supported in the analyses of both plastid and nuclear data. There are no supported conflicts in the relationships indicated by the different genomic compartments. *Isoetes wormaldii* is well supported as a highly divergent sister to all remaining *Isoetes*. From a sequence‐divergence perspective, the average median‐node depth to the *Isoetes* crown group increases by a factor of 2.55 (plastid data) or 1.95 (nuclear rDNA data) when *I. wormaldii* is included. Relationships among major groups of *Isoetes* agree entirely with those reported previously by Larsén and Rydin (2016) and by Larsén et al. (2022). Austro‐Asian Clade D is grouped sister to New World/American Clade E (BPP = 1.00); *I. malinverniana* (Clade C) is sister to this Clade D–E (BPP = 1.00); the nearly worldwide Clade B is sister to Clade C–E (BPP = 1.00); and the Gondwanan Clade A is sister to Clade B–E (BPP = 1.00). In Clade A, the African species *I. welwitschii* (Tanzania), *I. schweinfurthii* (Namibia), and *I. nigritiana* (Cameroon) group together. Sister to this African group are two Central American species—*I. pallida* (Mexico) and *I. cubana* (Mexico), and sister to this African‐American clade is *I. australis* from Australia. In Clade B, two samples of the Mediterranean species *I. durieui* (from France and Turkey, respectively) group together, and they are sister to an African clade comprising *I. transvaalensis* (South Africa), *I. giessii* (Namibia), *I. biafrana* (C. Afr. Rep.), and *I. aequinoctialis* (Zambia). In Clade D, *I. philippinensis* (Philippines) group with *I. neoguineensis* (Papua New Guinea), and they are sister to a clade comprising *I. drummondii* (Australia) sister to *I. sampathkumaranii* (India) and *I. pusilla* (Australia). In Clade E, only plastid data resolve the relationships among species. Two American species, *I. flaccida* and *I. occidentalis*, group with *I. weberi* from Brazil and *I. lechleri* from Bolivia as successive sisters. This last relationship is poorly supported. Sister to these four species is a clade comprising *I. azorica* from the Azores and *I. echinospora* from Canada (Figure 1; Appendix S2, Figures S1, S2).

#### Relaxed‐clock analyses

**Figure 2:**
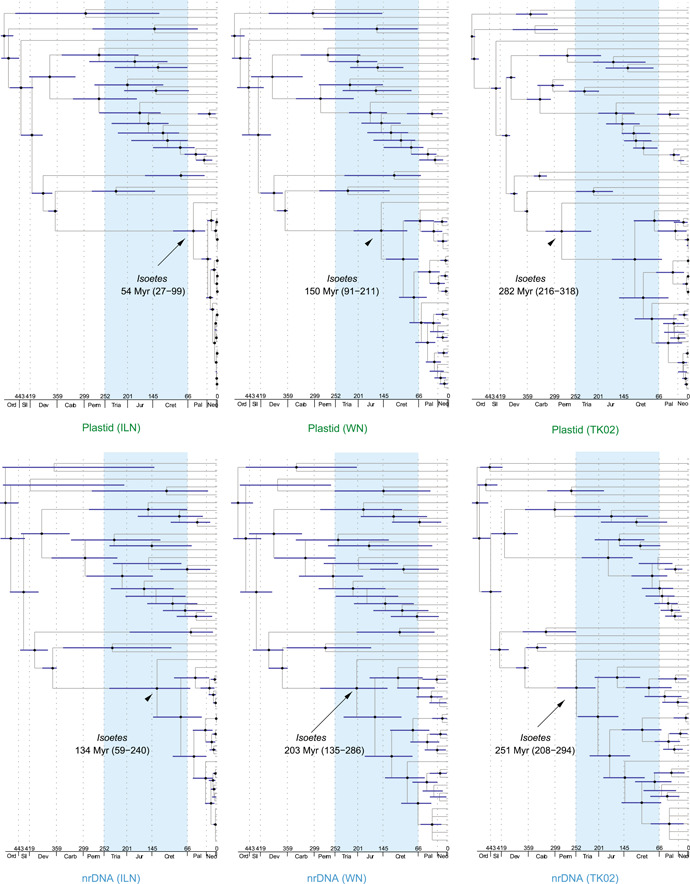
Schematic chronograms resulting from the analyses of plastid data (above) or nuclear ribosomal cistron data (below) and using the independent lognormal clock model (ILN, Drummond et al., 2006) (left column), the white noise clock model (WN, LePage et al., 2007) (middle column), and the Brownian motion clock model (TK02, Thorne and Kishino, 2002) (right column). The age estimate for crown group *Isoetes* is indicated for each analysis. Nodes indicated by a black dot are well supported and have a Bayesian posterior probability (BPP) of 0.95 or more (Alfaro et al., 2003; Erixon et al., 2003). The 95% HPD confidence intervals are indicated for each node by blue bars, and the Mesozoic (Triassic, Jurassic, and Cretaceous) is shaded in light blue.

Schematic chronograms resulting from the different relaxed‐clock analyses are reported in Figure 2 with the crown group age estimates of *Isoetes* indicated. Detailed versions of the chronograms are reported in Appendix S2 (Figures S3–S8), and specific age estimates for all nodes are given in Appendix S3 (plastid data) and Appendix S4 (nuclear rDNA data).

The crown group age estimates of *Isoetes* differed extensively depending on the source of the data (plastid vs. nuclear), but even more so depending on what relaxed‐clock model we used in our analyses. The median age estimate for the *Isoetes* crown group ranged between 54 Myr (ILN) and 282 Myr (TKO2) in the plastid‐based analyses and between 134 Myr (ILN) and 251 Myr (TK02) in the analyses of nuclear rDNA data. The 95% upper HPD limit (younger age) ranged between 27 Myr (ILN) and 216 Myr (TK02) in the plastid‐based analyses, and between 59 Myr (ILN) and 208 Myr (TK02) in the analyses of nuclear rDNA data. The corresponding lower HPD limit (older age) ranged between 99 Myr (ILN) and 318 Myr (TK02) in the plastid‐based analyses, and between 240 Myr (ILN) and 294 Myr (TK02) in the analyses of nuclear rDNA data (Figure 2; Appendix S2, Figures S3–S8).

## DISCUSSION

### Age estimates were inconsistent

Node ages in *Isoetes* have been investigated before, with strongly deviating results. Authors of recent work based on genomic data have argued that the living clade of *Isoetes* is young (compared with the ancient origin of the isoetalean lineage), i.e., from the Cenozoic (mid‐Paleogene to Neogene; Wood et al., 2020; Pereira et al., 2021). These results stand in contrast to those of earlier work that indicated much older (latest Paleozoic to mid‐Mesozoic) age estimates for the *Isoetes* crown group (Kim and Choi, 2016; Larsén and Rydin, 2016; Pereira et al., 2017). In agreement with Wood et al. (2020), we show that node age estimates may vary considerably between results retrieved from plastome data and nuclear data. We also show that the choice of clock model has a strong impact and is imperative for the resulting node ages. Our results, based on plastome and nuclear ribosomal cistron data from the same samples, show median age estimates for the crown group *Isoetes* that range from 282 Ma (mid‐Permian of the Paleozoic) to 54 Ma (earliest Eocene of the Cenozoic) depending on data and choice of clock model (Figure 2; Appendix S2, Figures S3–S8). Only under one specific condition (plastome data, analyzed with the ILN clock model) does the crown group age fall within the (early) Cenozoic. All results based on nuclear rDNA data, and all results using the WN and TK02 clock models result in much older age estimates for the crown group *Isoetes*, i.e., late Paleozoic to mid‐Mesozoic ages (Figure 2; Appendix S2, Figures S4–S8).

In passing, we note that in contrast to the deviating results on the age of the crown group, the stem age of *Isoetes* (i.e., the divergence between the isoetalean and selaginellalean lineages) consistently falls in the Devonian in our study and in previous work (Larsén and Rydin, 2016; Pereira et al., 2017; Wood et al., 2020; Pereira et al., 2021; Su et al., 2021). However, this apparent consistency among results and its correlation with the documented diverse representation of the isoetalean lineage that extends at least to the Late Devonian (see summary, e.g., of Larsén and Rydin, 2016) is governed by the fact that most of these studies have placed a similar age prior on this particular node, based on fossil information.

### Choice of clock model

We were particularly interested in the impact of one important component in dating analyses: the choice of clock model. We therefore analyzed the same two data sets with three clock models implemented in MrBayes that vary in how they relax the assumption of rate homogeneity among branches: the independent lognormal (ILN) model (Drummond et al., 2006), in which uncorrelated rates are drawn from a lognormal distribution, the white noise (WN) model (LePage et al., 2007), which is a continuous uncorrelated model of rate variation across lineages, and the Thorne–Kishino 2002 (TK02) model (Thorne and Kishino, 2002), which in contrast relies on the concept of autocorrelation of rates at different branches and assumes that relative rates change along branches as a random walk on a log scale starting at the root. The autocorrelated TK02 model yields the oldest median age estimates of the *Isoetes* crown group, which are relatively similar for both data sets (mid‐Permian, 282 Myr, based on plastid data and Permian‐Triassic border, 251 Myr, for nuclear rDNA data). The uncorrelated WN model results in slightly younger and slightly more divergent median node ages for the *Isoetes* crown group where the oldest estimate instead comes from nuclear data (latest Triassic, 203 Myr, based on nuclear rDNA data and latest Jurassic, 150 Myr based on plastid data). The uncorrelated ILN model yields the youngest as well as a much older median age estimate, thus resulting in the largest difference between data sets (earliest Cretaceous, 134 Ma, based on nuclear rDNA data, and earliest Eocene, 54 Ma, based on plastid data).

Node age estimation is a complicated and unconventional statistical problem where the parameters divergence time and molecular rate have to be estimated simultaneously from the branch length data, a task that is challenging also in a Bayesian framework (dos Reis and Yang, 2013). It is beyond the scope of this study to go into detail about the properties of clock models and precise reasons for the deviating results retrieved in the present study. We note, however, that based on sheer consistency of results, the plastid data performed worse than the nuclear rDNA data, and the uncorrelated ILN model returned the least‐consistent results and the autocorrelated TK02 the most‐consistent results. It has been repeatedly shown that node age estimation is highly sensitive to the relaxation model used (e.g., Ho et al., 2005; LePage et al., 2007; Battistuzzi et al., 2010; Crisp et al., 2014; Ho, 2014). For example, LePage et al. (2007) concluded based on general comparisons of a set of relaxed‐clock models that autocorrelated models significantly outperform uncorrelated models (unless the number of included taxa is small, 10 in their study) and that autocorrelation is an important component of temporal and evolutionary rate variation. Further, Crisp et al. (2014) and Worobey et al. (2014) demonstrated a very commonly used uncorrelated clock model as ill‐performing, consistently underestimating node ages and/or getting the (known) tree topology wrong. Translating these conclusions into the specific conditions of our study would suggest that the autocorrelated TK02 clock model is the best choice of the three we used, and thus that our oldest age estimates, those obtained with that model (median ages of 282–251 Myr for the *Isoetes* crown group), are the more reliable. On the other hand, the mode in which clock rates have evolved and should be modeled are rarely known a priori in empirical studies and are difficult to detect (Battistuzzi et al., 2010). Clock models with uncorrelated rates may for example fit empirical data sets better if there are substantial differences (rate changes) between adjacent branches. Further, for groups such as *Isoetes* (and *Ephedra*, Gnetales; Rydin et al., 2021) with a “broom‐like” tree appearance (long stem, short crown) and a long evolutionary distance to outgroups, it seems probable that different clock models would fit different parts of the tree best, indicating that local clocks may be a better fit. The random local clock model (RLC) implemented in software BEAST (Drummond and Suchard, 2010; Drummond et al., 2012) uses Bayesian stochastic search variable selection to infer whether a branch inherited its substitution rate from its parent (Ho and Duchêne, 2014), and only a small number of local clocks are typically needed to describe rate variation (Drummond and Suchard, 2010; Ho and Duchêne, 2014). The better fit of a local clock model for analyses of “broom‐like” clades has been shown in a study using simulated and real data (Crisp et al., 2014), where the RLC model correctly estimated the crown age of broom clades, whereas the uncorrelated lognormal clock also implemented in BEAST (and used by Wood et al., 2020), consistently underestimated the age of the crown group (Crisp et al., 2014). We used the random local clock (RLC) model in a previous study of node ages in *Isoetes* (Larsén and Rydin, 2016), but dismissed the results of that analysis for several reasons. The model provided significantly worse fit to data than other clock models we used and produced some spurious topological results not seen in any other study (Larsén and Rydin, 2016). Further, the RLC model is apparently computationally demanding and requires extremely long runs to reach convergence (Larsén and Rydin, 2016), a problem seen by others (Bellot and Renner, 2014). A local clocks model was therefore not used in the present study.

### Comparison with results in previous work

Clearly, the analytical approaches taken in previous studies of node age estimation in *Isoetes* differ in several respects, and results from different studies are rarely completely comparable. Despite complicating factors, our aim with the present study was to evaluate, at least to the extent possible, the strongly deviating age estimates of the crown group *Isoetes* that exist in the literature. We were particularly interested in those found in recent work based on phylogenomic data (Wood et al., 2020; Pereira et al., 2021). We note, however, that the amount of included data may be of limited importance; other factors (most of all calibration to absolute time but also choice of clock model and perhaps taxon sampling density) are more important (Ho and Duchêne, 2014). Several studies have indicated little error in, for example, branch length estimation, also with data sets of moderate size (e.g., Thorne and Kishino, 2002; dos Reis and Yang, 2013). Early studies on node age estimation in *Isoetes* that were based on less than a handful of molecular markers (Kim and Choi, 2016; Larsén and Rydin, 2016; Pereira et al., 2017) may thus be of value since these studies include a much more complete species representation of the ingroup (but see below on the debated importance of taxon sampling density). Outgroup sampling has varied in these studies, from *Selaginella* only (Kim and Choi, 2016), to lycopods only (Pereira et al., 2017; Pereira et al., 2021) to inclusion of representatives from all major groups of land plants (Larsén and Rydin, 2016; Wood et al., 2020).

#### Taxon sampling

It has long been recognized that taxon sampling strategies affect phylogenetic reconstruction (Pollock et al., 2002; Rydin and Källersjö, 2002; Zwickl and Hillis, 2002), including node age estimates (Linder et al., 2005; Schulte, 2013) for which the taxon sampling density should be considered during the a priori process of selecting suitable clock model(s) (LePage et al., 2007; Ho and Duchêne, 2014). However, the opposite has also been argued, i.e., that taxon sampling (and data size in general) has limited impact on topology and node age estimates (e.g., Rosenberg and Kumar, 2001; Morris et al., 2018). It has even been specifically demonstrated that sparse taxon sampling has little impact on node age estimation in *Isoetes* (Wood et al., 2020). Not least considering the latter finding, it seems unlikely that results on node ages in *Isoetes* retrieved in studies using a relatively restricted sample of taxa in the genus (Wood et al., 2020; Pereira et al., 2021, and the present study) would be less reliable compared with studies employing denser sampling. Importantly, however, no previous study that addressed node ages in *Isoetes* has included *Isoetes wormaldii*, recently shown to be the highly divergent sister species of the remaining *Isoetes* (Larsén et al., 2022). The inclusion of the genetically (and morphologically) distinct/distant *Isoetes wormaldii*, thus falling outside of what was previously thought to be the crown group, will undoubtedly have a strong and direct impact on the age estimate of the living clade (Larsén et al., 2022), and indeed, our average median age of the *Isoetes* crown group increased by a factor of 2.55 (plastid data) and by 1.95 (nuclear rDNA data) when *I. wormaldii* was added to our data set. However, it should be noted that our mostly much older age estimates of the crown group *Isoetes* compared with those of Wood et al. (2020) and Pereira et al. (2021) cannot be explained solely by the inclusion of *Isoetes wormaldii*. They remain also when comparing with the estimated ages of our node 11, excluding *Isoetes wormaldii* (Appendix S2, Figures S4–S8). Only in one case (our analysis of plastid data using the ILN clock model; Appendix S2, Figure S3) is our result comparable to that of the corresponding analyses of plastid data of Wood et al. (2020) and Pereira et al. (2021).

#### Data sampling

Wood et al. (2020) thus also based their work on plastid genomic data and nuclear (ortholog) data, with resulting node ages retrieved from plastid data being distinctly younger than those estimated based on nuclear data (Wood et al., 2020). The analyses of plastid genomic data in Pereira et al. (2021) resulted in a similar Neogene age for the *Isoetes* crown group (around 20 Ma) as did those of Wood et al. (2020). While Pereira et al. (2021) argued that plastid data are suitable for node age estimation because of uniparental inheritance and lack of recombination (not always true though, see e.g., Sullivan et al., 2017), Wood et al. (2020) argued that plastid data are not suitable for node age estimation in *Isoetes* because of high rate variability (between the crown group *Isoetes* and its stem) in plastid data compared with nuclear data, and they based their conclusions on node ages in *Isoetes* on results from nuclear data. As assessed from branch lengths, we have not seen this in our results (Figure 1) (i.e., a higher rate variation between the crown group *Isoetes* and its stem in analyses based on plastid data compared with nuclear data). However, our results tentatively indicate that results of analyses of plastid data are more sensitive to the choice of clock model (at least in *Isoetes*). As in Wood et al. (2020), plastid data returned the youngest age estimate of the crown group *Isoetes* (54 Ma in our study, using the ILN clock model; Figure 2: top left), but using several alternative clock models, we show that the same plastid data set also provide the oldest age estimate in our study (282 Myr, using the TK02 clock model; Figure 2: top right). The difference in node age estimates is smaller for nuclear rDNA data (Figure 2), although still distinct and pronounced; the age of the *Isoetes* crown group as estimated based on nuclear rDNA data ranges from 134 Myr (ILN clock model) to 251 Myr (TK02 model) (Figure 2).

#### Calibration to absolute time

There is ample argumentation in the literature that the calibration to absolute time is the single most important component in analyses of divergence times of clades based on molecular data and is potentially an important source of error in node age estimation in empirical work (e.g., Sauquet et al., 2012; Crisp et al., 2014; Duchêne et al., 2014; Hipsley and Müller, 2014; Ho and Duchêne, 2014). It has been argued that it is important to include several calibrations, preferably close to the root of the phylogeny (Duchêne et al., 2014), but lack of calibrations in the ingroup may also pose problems, in particular for long‐stemmed “broom” clades (Crisp et al., 2014; Rydin et al., 2021) such as *Isoetes*. Despite the importance of calibration, we have not focused on calibration strategies in the present study because most studies of node ages in *Isoetes* have used a similar and appropriate approach, making the most out of the fossil record as currently described and understood. As in several previous studies (e.g., Larsén and Rydin, 2016; Wood et al., 2020; Pereira et al., 2021), we have employed a conservative approach to fossil calibration that follows recommendations for best practice (Parham et al., 2012). We do not accept any isoetalean fossil as suitable for calibration of the crown group *Isoetes* or within it because no fossil has to date been indisputably placed phylogenetically at or within the crown group *Isoetes* (see discussions by Larsén and Rydin, 2016; Wood et al., 2020 for justification). Further, to enhance comparison of results, we here followed the method of Wood et al. (2020) regarding calibration to absolute time, using only two calibration points: the root node (i.e., the origin of land plants based on Ordovician cryptospores) and the split between the isoetalean and selaginellalean lineages based on the earliest‐known rhizomorphic lycopsids from the Late Devonian. Additional calibration points within *Isoetes* would clearly be beneficial in future studies but would require that new fossils and/or a better understanding of fossils provide the necessary information. Such information is currently unavailable but may be available soon (from research in progress by coauthors E. Larsén and C. Rydin). Or, alternatively, other sources of calibration might be found and used (as discussed, e.g., by Hipsley and Müller [2014]), as a way forward for groups with a fossil record that is insufficient for calibration.

## CONCLUSIONS

Recent studies of node ages in *Isoetes* have argued that the clade comprising all living species is young compared with the ancient ancestry of the lineage, i.e., from the mid‐Paleogene to Neogene (Wood et al., 2020; Pereira et al., 2021). We find no consistent support for such a Cenozoic age of the *Isoetes* crown group. However, there is no consistent support for any other age either. Using two same‐sample genomic data sets (plastid data and nuclear ribosomal cistron data), we obtained seemingly well‐founded, yet contradictory, results on node age estimations simply by analyzing the data using different clock models. Other settings, choices, parameters and aspects of the evolutionary model will also influence absolute age estimates (such as sampling of taxa and data, prior settings, selection of substitution model and calibration points, e.g., LePage et al., 2007), but here all other aspects are the same across all our analyses and cannot explain the deviating results we observe.

The resulting median ages of the crown group *Isoetes* ranged from 282 Myr (plastid data, autocorrelated TK02 clock model) to 54 Myr (plastid data, uncorrelated ILN clock model), a difference of nearly 230 Myr. The effect was most severe for the plastid data, but almost as prominent for the nuclear rDNA data. The Cenozoic (mid‐Paleogene) median age concluded by Wood et al. (2020) for the *Isoetes* crown group was only reproduced in our study under one specific condition, i.e., using plastid data and the uncorrelated ILN clock model. All our analyses of the nuclear cistron data set as well as analyses of the plastid data set using other clock models yielded latest Paleozoic to Mesozoic age estimates for the *Isoetes* crown group. The Neogene median age (approximately 20 Myr) found by Wood et al. (2020) and Pereira et al. (2021) based on plastid data was never reproduced in any of our analyses that included *Isoetes wormaldii*. For simplicity, we chose to use median ages as the basis for our discussion, and we note that confidence intervals were relatively broad in all cases. Considering confidence intervals instead of median ages, the age estimates spanned more than 290 Myr depending on data type and clock model. Pereira et al. (2021) pointed out that their confidence intervals on node ages in *Isoetes* are attractively narrow (as e.g., indicated in their fig. 5), much narrower than those of most previous studies of node ages in *Isoetes* (e.g., Larsén and Rydin, 2016; Wood et al., 2020), and they argued that their (more extensive) data partitioning was advantageous and led to narrower confidence intervals (Pereira et al., 2021). While we recognize that their extensive data partitioning may indeed have led to narrower confidence intervals, the risk for over‐parameterization and/or results that are only valid under certain specific conditions must also be considered.

More research is needed regarding general aspects of clock model choice and its consequences (Ho and Duchêne, 2014; Douglas et al., 2021). In empirical studies, accuracy of results from analyses retrieved using different models selected a priori according to criteria presumably relevant for the data at hand can be assessed relatively simply, as we have done here, by reflecting on their consistency (e.g., Battistuzzi et al., 2010). The same can also be done statistically and more explicitly, for example, by using Bayes factors and stepping stone sampling. However, even the “best fitting” model can in fact have a poor fit to the data at hand. For example, in their study of avian virus data, Worobey et al. (2014) argued that any method that does not allow for host‐specific local clocks may produce spurious and unreliable results. Estimating node ages in long‐stemmed clades (“broom‐like” clades) can be particularly problematic (Crisp et al., 2014; Rydin et al., 2021), and the stem lineage of *Isoetes* is still relatively long compared with branches in the crown, despite the fact that the stem was broken up by the inclusion of the genetically divergent *Isoetes wormaldii*, recently discovered to be sister to the remaining genus (Larsén et al., 2022).

Evolutionary models are constantly being produced and improved, but there are many components involved in node age estimation and difficulties in reaching a reasonable consensus have been demonstrated before, both in empirical studies and using data simulation (e.g., Battistuzzi et al., 2010; Sauquet et al., 2012; dos Reis and Yang, 2013; Crisp et al., 2014; Testo et al., 2018; Rydin et al., 2021). It will likely be challenging also in the future to find models that can handle the long evolutionary distances and extreme rate heterogeneity among the major lineages of heterosporous lycopods (see also Figure 1).

We refrain from stating whether a crown group median age of 54 Myr or 282 Myr (134 or 251 for nuclear rDNA data) is the best estimate. Clearly, the choice of data and clock model strongly influences node age estimates in *Isoetes*, even to an extent when resulting dates are so far apart that using them for interpretations of the evolution of the group becomes pointless. We conclude, however, that there is no consistent support for a Cenozoic age of the crown group *Isoetes*, as suggested in recent work (Wood et al., 2020; Pereira et al., 2021). Most of our results, and all those obtained with data and clock models suggested in the literature to perform better (nuclear data; autocorrelated clock models) return a latest Paleozoic to Mesozoic median age for the *Isoetes* crown group. As assessed from recommendations in the methodological literature, the most important and straightforward improvement to consider in future studies to reach the goal of a coherent and accurate age estimate for the crown group *Isoetes* should be to use additional calibrations to absolute time. Currently, the necessary information for doing that is lacking, but ongoing studies (by coauthors E. Larsén and C. Rydin) appear promising regarding an improved understanding of certain fossil information.

## AUTHOR CONTRIBUTIONS

N.W, E.L., and C.R. designed the research. E.L. and A.K. conducted laboratory work. N.W. and E.L. assembled and analyzed the data. All authors interpreted the results. N.W. and C.R. wrote the manuscript with comments from E.L. and A.K. All authors read and approved the final version of the text. The authors have no conflicts of interest to declare.

## Supporting information


**Appendix S1**. List of lab identification numbers, taxon names, taxon distribution, DNA voucher information and GenBank accession numbers for sequences used in the study.

**Appendix S2**. Phylograms and chronograms resulting from non‐clock and relaxed‐clock analyses of plastid data and nuclear ribosomal cistron data.

**Appendix S3**. Detailed results from the individual divergence time analyses using complete plastid CDS data and alternative relaxed‐clock models (ILN, WN, and TK02; see text).

**Appendix S4**. Detailed results from the individual divergence time analyses using nuclear ribosomal cistron data and alternative relaxed‐clock models (ILN, WN, and TK02; see text)

## Data Availability

Newly produced sequence data are deposited at GenBank (for accessions, see Appendix S1). All data sets and the supporting information (see below) are permanently archived at the Dryad Digital Repository: https://doi.org/10.5061/dryad.q2bvq83pc (Wikström et al., 2022).

## References

[ajb216108-bib-0001] Alfaro, M. E. , S. Zoller , and F. Lutzoni . 2003. Bayes or bootstrap? A simulation study comparing the performance of Bayesian Markov chain Monte Carlo sampling and bootstrapping in assessing phylogenetic confidence. Molecular Biology and Evolution 20: 255–266. 12598693 10.1093/molbev/msg028

[ajb216108-bib-0002] Arrigo, N. , J. Therrien , C. L. Anderson , M. D. Windham , C. H. Haufler , and M. S. Barker . 2013. A total evidence approach to understanding phylogenetic relationships and ecological diversity in *Selaginella* subg. *Tetragonostachys* . American Journal of Botany 100: 1672–1682. 23935110 10.3732/ajb.1200426

[ajb216108-bib-0003] Ash, S. R. 1972. Late Triassic plants from the Chinle Formation in north‐eastern Arizona. Palaeontology 15: 598–618.

[ajb216108-bib-0004] Ash, S. R. , and K. B. Pigg . 1991. A new Jurassic *Isoetites* (Isoetales) from the Wallowa Terrane in Hells Canyon, Oregon and Idaho. American Journal of Botany 78: 1636–1642.

[ajb216108-bib-0005] Baker, J. G. 1880. A synopsis of the species of *Isoetes* . Journal of Botany, British and Foreign 18: 65‐70, 105–110.

[ajb216108-bib-0006] Banks, H. P. , P. M. Bonamo , and J. D. Grierson . 1972. *Leclercqia complexa* gen. et sp. nov., a new lycopod from the late Middle Devonian of eastern New York. Review of Palaeobotany and Palynology 14: 19–40.

[ajb216108-bib-0007] Bateman, R. M. 1992. Morphometric reconstruction, palaeobiology and phylogeny of *Oxroadia gracilis* Alvin emend. and *O. conferta* sp. nov.: anatomically preserved rhizomorphic lycopsids from the Dinantian of Oxroad Bay, SE Scotland. Palaeontographica B 228: 29–103.

[ajb216108-bib-0008] Bateman, R. M. , and W. A. DiMichele . 1994. Heterospory: the most iterative key innovation in the evolutionary history of the plant kingdom. Biological Reviews 69: 345–417.

[ajb216108-bib-0009] Battistuzzi, F. U. , A. Filipski , S. B. Hedges , and S. Kumar . 2010. Performance of relaxed‐clock methods in estimating evolutionary divergence times and their credibility intervals. Molecular Biology and Evolution 27: 1289–1300. 20093431 10.1093/molbev/msq014 PMC2877995

[ajb216108-bib-0010] Bek, J. , M. Libertín , and J. Drábková . 2009. *Selaginella labutae* sp. nov., a new compression herbaceous lycopsid and its spores from the Kladno‐Rakovník Basin, Bolsovian of the Czech Republic. Review of Palaeobotany and Palynology 155: 101–115.

[ajb216108-bib-0011] Bellot, S. , and S. S. Renner . 2014. Exploring new dating approaches for parasites: the worldwide Apodanthaceae (Cucurbitales) as an example. Molecular Phylogenetics and Evolution 80: 1–10. 25057774 10.1016/j.ympev.2014.07.005

[ajb216108-bib-0012] Bolin, J. F. , R. D. Bray , and L. J. Musselman . 2011. A new species of diploid quillwort (*Isoetes*, Isoetaceae, Lycophyta) from Lebanon. Novon 21: 295–298, 4.

[ajb216108-bib-0013] Bonfield, J. K. , and A. Whitwham . 2010. Gap5‐editing the billion fragment sequence assembly. Bioinformatics 26: 1699–1703. 20513662 10.1093/bioinformatics/btq268 PMC2894512

[ajb216108-bib-0014] Brown, R. C. , B. E. Lemmon , M. Shimamura , J. C. Villarreal , and K. S. Renzaglia . 2015. Spores of relictual bryophytes: diverse adaptations to life on land. Review of Palaeobotany and Palynology 216: 1–17.

[ajb216108-bib-0015] Burnard, D. , L. Shepherd , L. Perrie , and A. Munkacsi . 2016. Phylogenetic relationships of New Zealand Lycopodiaceae. Plant Systematics and Evolution 302: 661–667.

[ajb216108-bib-0016] Chen, D.‐K. , X.‐M. Zhou , C. J. Rothfels , L. D. Shepherd , R. Knapp , L. Zhang , N. T. Lu , et al. 2022. A global phylogeny of Lycopodiaceae (Lycopodiales; lycophytes) with the description of a new genus, *Brownseya*, from Oceania. Taxon 71: 25–51.

[ajb216108-bib-0017] Cox, P. A. , and R. J. Hickey . 1984. Convergent megaspore evolution and *Isoetes* . American Naturalist 124: 437–441.

[ajb216108-bib-0018] Cressler, W. L , and H. W. Pfefferkorn . 2005. A Late Devonian isoetalean lycopsid, *Otzinachsonia beerboweri*, gen. et sp. nov., from north‐central Pennsylvania, USA. American Journal of Botany 92: 1131–1140. 21646135 10.3732/ajb.92.7.1131

[ajb216108-bib-0019] Crisp, M. D. , N. B. Hardy , and L. G. Cook . 2014. Clock model makes a large difference to age estimates of long‐stemmed clades with no internal calibration: a test using Australian grasstrees. BMC Evolutionary Biology 14: 263. 25523814 10.1186/s12862-014-0263-3 PMC4279595

[ajb216108-bib-0020] Dettmann, M. E. 1986. Early Cretaceous palynoflora of subsurface strata correlative with the Koonwarra Fossil Bed, Victoria. *In* P. A. Jell and J. Roberts [eds.], Plants and invertebrates from the Lower Cretaceous Koonwarra Fossil Beds, South Gippsland, Victoria, 79–110. Association of Australasian Palaeontologists, Sydney, Australia.

[ajb216108-bib-0021] DiMichele, W. A. , and R. M. Bateman . 1996. The rhizomorphic lycopsids: a case‐study in paleobotanical classification. Systematic Botany 21: 535–552.

[ajb216108-bib-0022] DiMichele, W. A. , R. M. Bateman , G. W. Rothwell , I. A. P. Duijnstee , S. D. Elrick , and C. V. Looy . 2022. *Stigmaria*: a review of the anatomy, development, and functional morphology of the rootstock of the arboreous Lycopsids. International Journal of Plant Sciences 183: 493–534.

[ajb216108-bib-0023] dos Reis, M. , and Z. Yang . 2013. The unbearable uncertainty of Bayesian divergence time estimation. Journal of Systematics and Evolution 51: 30–43.

[ajb216108-bib-0024] Douglas, J. , R. Zhang , and R. Bouckaert . 2021. Adaptive dating and fast proposals: revisiting the phylogenetic relaxed clock model. PLoS Computational Biology 17: e1008322. 33529184 10.1371/journal.pcbi.1008322 PMC7880504

[ajb216108-bib-0025] Doyle, J. J. 1991. DNA protocols for plants. *In* G. Hewitt , A. W. B. Johnson , and J. P. W. Young [eds.], Molecular techniques in taxonomy, 283–293. Springer, Berlin, Germany.

[ajb216108-bib-0026] Doyle, J. J. , and J. L. Doyle . 1987. A rapid DNA isolation procedure for small quantities of fresh leaf tissue. Phytochemical Bulletin 19: 11–15.

[ajb216108-bib-0027] Drummond, A. J. , S. Y. W. Ho , M. J. Phillips , and A. Rambaut . 2006. Relaxed phylogenetics and dating with confidence. PLoS Biology 4: e88. 16683862 10.1371/journal.pbio.0040088 PMC1395354

[ajb216108-bib-0028] Drummond, A. J. , and M. A. Suchard . 2010. Bayesian random local clocks, or one rate to rule them all. BMC Biology 8: 114. 20807414 10.1186/1741-7007-8-114 PMC2949620

[ajb216108-bib-0029] Drummond, A. J. , M. A. Suchard , X.‐P. Dong , and A. Rambaut . 2012. Bayesian phylogenetics with BEAUti and the BEAST 1.7. Molecular Biology and Evolution 29: 1969–1973. 22367748 10.1093/molbev/mss075 PMC3408070

[ajb216108-bib-0030] Duchêne, S. , R. Lanfear , and S. Y. W. Ho . 2014. The impact of calibration and clock‐model choice on molecular estimates of divergence times. Molecular Phylogenetics and Evolution 78: 277–289. 24910154 10.1016/j.ympev.2014.05.032

[ajb216108-bib-0031] Edgar, R. C. 2004. MUSCLE: multiple sequence alignment with high accuracy and high throughput. Nucleic Acids Research 32: 1792–1797. 15034147 10.1093/nar/gkh340 PMC390337

[ajb216108-bib-0032] Edwards, D. , and P. Kenrick . 2015. The early evolution of land plants, from fossils to genomics: a commentary on Lang (1937) ‘On the plant‐remains from the Downtonian of England and Wales’. Philosophical Transactions of the Royal Society, B, Biological Sciences 370: 20140343. 10.1098/rstb.2014.0343 PMC4360123 25750238

[ajb216108-bib-0033] Engelmann, G. 1882. The genus *Isoetes* in North America. Transactions of the Academy of Science of St. Louis 4: 358–390.

[ajb216108-bib-0034] Erixon, P. , B. Svennblad , T. Britton , and B. Oxelman . 2003. Reliability of Bayesian posterior probabilities and bootstrap frequences in phylogenetics. Systematic Biology 52: 665–673. 14530133 10.1080/10635150390235485

[ajb216108-bib-0035] Field, A. R. , W. Testo , P. D. Bostock , J. A. M. Holtum , and M. Waycott . 2016. Molecular phylogenetics and the morphology of the Lycopodiaceae subfamily Huperzioideae supports three genera: *Huperzia, Phlegmariurus* and *Phylloglossum* . Molecular Phylogenetics and Evolution 94: 635–657. 26493224 10.1016/j.ympev.2015.09.024

[ajb216108-bib-0036] Freund, F. D. , W. A. Freyman , and C. J. Rothfels . 2018. Inferring the evolutionary reduction of corm lobation in *Isoetes* using Bayesian model‐averaged ancestral state reconstruction. American Journal of Botany 105: 275–286. 29573405 10.1002/ajb2.1024

[ajb216108-bib-0037] Fuchs‐Eckert, H. P. 1982. Zur heutigen kenntnis von Vorkommen und Verbreitung der sudamericanischen *Isoetes*‐arten. Proceedings of the Koninklijke Nederlandse Akademie van Wetenschappen C85: 205–260.

[ajb216108-bib-0038] Fučíková, K. , P. O. Lewis , and L. A. Lewis . 2016. Chloroplast phylogenomic data from the green algal order Sphaeropleales (Chlorophyceae, Chlorophyta) reveal complex patterns of sequence evolution. Molecular Phylogenetics and Evolution 98: 176–183. 26903036 10.1016/j.ympev.2016.01.022

[ajb216108-bib-0039] Gensel, P. G. 2008. The earliest land plants. Annual Review of Ecology, Evolution, and Systematics 39: 459–477.

[ajb216108-bib-0040] Gensel, P. G. , and V. M. Albright . 2006. *Leclercqia complexa* from the Early Devonian (Emsian) of northern New Brunswick, Canada. Review of Palaeobotany and Palynology 142: 103–121.

[ajb216108-bib-0041] Gerrienne, P. , B. Cascales‐Minana , C. Prestianni , P. Steemans , and L. Cheng‐Sen . 2018. *Lilingostrobus chaloneri* gen. et sp. nov., a Late Devonian woody lycopsid from Hunan, China. PLoS One 13: e0198287. 29995908 10.1371/journal.pone.0198287 PMC6050970

[ajb216108-bib-0042] Gouy, M. , S. Guindon , and O. Gascuel . 2009. SeaView version 4: A multiplatform graphical user interface for sequence alignment and phylogenetic tree building. Molecular Biology and Evolution 27: 221–224. 19854763 10.1093/molbev/msp259

[ajb216108-bib-0043] Gray, J. 1985. The microfossil record of early land plants: advances in understanding of early terrestrialization, 1970–1984. Philosophical Transactions of the Royal Society of London B 309: 167–195. 10.1098/rstb.2000.0612 PMC1692785 10905606

[ajb216108-bib-0044] Guindon, S. , J. F. Dufayard , V. Lefort , M. Anisimova , W. Hordijk , and O. Gascuel . 2010. New algorithms and methods to estimate maximum‐likelihood phylogenies: assessing the performance of PhyML 3.0. Systematic Biology 59: 307–321. 20525638 10.1093/sysbio/syq010

[ajb216108-bib-0045] Guindon, S. , and O. Gascuel . 2003. A simple, fast, and accurate algorithm to estimate large phylogenies by maximum likelihood. Systematic Biology 52: 696–704. 14530136 10.1080/10635150390235520

[ajb216108-bib-0046] Harris, T. M. 1961. The Yorkshire Jurassic flora. I. Thallophyta‐Pteridophyta. British Museum of Natural History, London, UK.

[ajb216108-bib-0047] Herrera, F. , W. L. Testo , A. R. Field , E. G. Clark , P. S. Herendeen , P. R. Crane , and G. Shi . 2022. A permineralized Early Cretaceous lycopsid from China and the evolution of crown clubmosses. New Phytologist 233: 2310–2322. 34981832 10.1111/nph.17874

[ajb216108-bib-0048] Hetherington, A. J. , and L. Dolan . 2017. The evolution of lycopsid rooting structures: conservatism and disparity. New Phytologist 215: 538–544. 27901273 10.1111/nph.14324

[ajb216108-bib-0049] Hickey, R. J. 1986. The early evolutionary and morphological diversity of *Isoetes*, with descriptions of two new neotropical species. Systematic Botany 11: 309–321.

[ajb216108-bib-0050] Hickey, R. J. 1990. Studies of neotropical *Isoetes* L.: the subgenus *Euphyllum* Hickey, subg. nov. Annals of the Missouri Botanical Garden 77: 239–245.

[ajb216108-bib-0051] Hipsley, C. A. , and J. Müller . 2014. Beyond fossil calibrations: realities of molecular clock practices in evolutionary biology. Frontiers in Genetics 5: 138. 24904638 10.3389/fgene.2014.00138 PMC4033271

[ajb216108-bib-0052] Ho, S. Y. W. 2014. The changing face of the molecular evolutionary clock. Trends in Ecology & Evolution 29: 496–503. 25086668 10.1016/j.tree.2014.07.004

[ajb216108-bib-0053] Ho, S. Y. W. , and S. Duchêne . 2014. Molecular‐clock methods for estimating evolutionary rates and timescales. Molecular Ecology 23: 5947–5965. 25290107 10.1111/mec.12953

[ajb216108-bib-0054] Ho, S. Y. W. , M. J. Phillips , A. J. Drummond , and A. Cooper . 2005. Accuracy of rate estimation using relaxed‐clock models with a critical focus on the early Metazoan radiation. Molecular Biology and Evolution 22: 1355–1363. 15758207 10.1093/molbev/msi125

[ajb216108-bib-0055] Hoot, S. , W. C. Taylor , and N. S. Napier . 2006. Phylogeny and biogeography of *Isoetes* (Isoetaceae) based on nuclear and chloroplast DNA sequence data. Systematic Botany 31: 449–460.

[ajb216108-bib-0056] Hoot, S. B. , N. S. Napier , and W. C. Taylor . 2004. Revealing unknown or extinct lineages within *Isoëtes* (Isoëtaceae) using DNA sequences from hybrids. American Journal of Botany 91: 899–904. 21653446 10.3732/ajb.91.6.899

[ajb216108-bib-0057] Huelsenbeck, J. P. , and F. R. Ronquist . 2001. MRBAYES: Bayesian inference of phylogenetic trees. Bioinformatics 17: 754–755. 11524383 10.1093/bioinformatics/17.8.754

[ajb216108-bib-0058] Jackman, S. D. , B. P. Vandervalk , H. Mohamadi , J. Chu , S. Yeo , S. A. Hammond , G. Jahesh , et al. 2017. ABySS 2.0: resource‐efficient assembly of large genomes using a Bloom filter. Genome Research 27: 768–777. 28232478 10.1101/gr.214346.116 PMC5411771

[ajb216108-bib-0059] Jermy, A. C. 1990. Isoetaceae. *In* K. U. Kramer and P. S. Green [eds.], The families and genera of vascular plants, vol. I. Pteridophytes and gymnosperms, 26–31. Springer Verlag, Berlin, Germany.

[ajb216108-bib-0060] Ji, S.‐G. , K. K. Huo , J. Wang , and S. L. Pan . 2008. A molecular phylogenetic study of Huperziaceae based on chloroplast *rbcL* and *psbA‐trnH* sequences. Journal of Systematics and Evolution 46: 213–219.

[ajb216108-bib-0061] Karol, K. , K. Arumuganathan , J. Boore , A. Duffy , K. Everett , J. Hall , S. Hansen , et al. 2010. Complete plastome sequences of *Equisetum arvense* and *Isoetes flaccida*: implications for phylogeny and plastid genome evolution of early land plant lineages. BMC Evolutionary Biology 10: 321. 20969798 10.1186/1471-2148-10-321 PMC3087542

[ajb216108-bib-0062] Kenrick, P. 2003. Fishing for the first plants. Nature 425: 248–249. 13679900 10.1038/425248a

[ajb216108-bib-0063] Kenrick, P. , and P. R. Crane . 1997a. The origin and early diversification of land plants: a cladistic study. Smithsonian Institution Press, Washington, D.C., USA.

[ajb216108-bib-0064] Kenrick, P. , and P. R. Crane . 1997b. The origin and early evolution of plants on land. Nature 389: 33–39.

[ajb216108-bib-0065] Kent, J. W. 2002. BLAT—The BLAST‐like alignment tool. Genome Research 12: 656–664. 11932250 10.1101/gr.229202 PMC187518

[ajb216108-bib-0066] Kim, C. , and H.‐K. Choi . 2016. Biogeography of North Pacific *Isoetes* (Isoetaceae) inferred from nuclear and chloroplast DNA sequence data. Journal of Plant Biology 59: 386–396.

[ajb216108-bib-0067] Korall, P. , and P. Kenrick . 2002. Phylogenetic relationships in Selaginellaceae based on *rbcL* sequences. American Journal of Botany 89: 506–517. 21665649 10.3732/ajb.89.3.506

[ajb216108-bib-0068] Korall, P. , and P. Kenrick . 2004. The phylogenetic history of Selaginellaceae based on DNA sequences from the plastid and nucleus: extreme substitution rates and rate heterogeneity. Molecular Phylogenetics and Evolution 31: 852–864. 15120383 10.1016/j.ympev.2003.10.014

[ajb216108-bib-0069] Korall, P. , P. Kenrick , and J. Therrien . 1999. Phylogeny of Selaginellaceae: evaluation of generic/subgeneric relationships based on *rbcL* gene sequences. International Journal of Plant Sciences 160: 585–594.

[ajb216108-bib-0070] Kotyk, M. E. , J. F. Basinger , P. G. Gensel , and T. A. de Freitas . 2002. Morphologically complex plant macrofossils from the Late Silurian of Arctic Canada. American Journal of Botany 89: 1004–1013. 21665700 10.3732/ajb.89.6.1004

[ajb216108-bib-0071] Kranz, H. D. , and V. A. R. Huss . 1996. Molecular evolution of pteridophytes and their relationships to seed plants: evidence from complete 18S rRNA gene sequences. Plant Systematics and Evolution 202: 1–11.

[ajb216108-bib-0072] Lang, W. H. , and I. C. Cookson . 1935. On a flora, including vascular land plants, associated with *Monograptus*, in rocks of Silurian age, from Victoria, Australia. Philosophical Transactions of the Royal Society of London, B, Biological Sciences 224: 421–449.

[ajb216108-bib-0073] Larsén, E. , and C. Rydin . 2016. Disentangling the phylogeny of *Isoetes* (Isoetales), using nuclear and plastid data. International Journal of Plant Sciences 177: 157–174.

[ajb216108-bib-0074] Larsén, E. , N. Wikström , A. Khodabandeh , and C. Rydin . 2022. Phylogeny of Merlin's grass (Isoetaceae): revealing an “*Amborella* syndrome” and the importance of geographic distribution for understanding current and historical diversity. BMC Ecology and Evolution 22: 32. 35296231 10.1186/s12862-022-01988-w PMC8928685

[ajb216108-bib-0075] LePage, T. , D. H. P. Bryant , and N. Lartillot . 2007. A general comparison of relaxed molecular clock models. Molecular Biology and Evolution 24: 2669–2680. 17890241 10.1093/molbev/msm193

[ajb216108-bib-0076] Li, H. , and R. Durbin . 2009. Fast and accurate short read alignment with Burrows–Wheeler transform. Bioinformatics 25: 1754–1760. 19451168 10.1093/bioinformatics/btp324 PMC2705234

[ajb216108-bib-0077] Linder, H. P. , C. R. Hardy , and F. Rutschmann . 2005. Taxon sampling effects in molecular clock dating: an example from the African Restionaceae. Molecular Phylogenetics and Evolution 35: 569–582. 15878126 10.1016/j.ympev.2004.12.006

[ajb216108-bib-0078] Lindley, J. , and W. Hutton . 1833. The fossil flora of Great Britain. James Ridgway, London, UK.

[ajb216108-bib-0079] McLoughlin, S. , I.‐M. Jansson , and V. Vajda . 2014. Megaspore and microfossil assemblages reveal diverse herbaceous lycophytes in the Australian Early Jurassic flora. Grana 53: 22–53.

[ajb216108-bib-0080] Morris, J. L. , M. N. Puttick , J. W. Clark , D. Edwards , P. Kenrick , S. Pressel , C. H. Wellman , et al. 2018. The timescale of early land plant evolution. Proceedings of the National Academy of Sciences, USA 115: E2274–E2283. 10.1073/pnas.1719588115 PMC5877938 29463716

[ajb216108-bib-0081] Motelay, L. , and A. Vendryès . 1882. Monographie des Isoëteæ. Actes de la Société Linnéenne de Bordeaux 36: 309–404.

[ajb216108-bib-0082] Nylander, J. A. A. 2004. MrAIC.pl. Program distributed by the author. Evolutionary Biology Centre, Uppsala University, Uppsala, Sweden.

[ajb216108-bib-0083] One Thousand Plant Transcriptomes Initiative . 2019. One thousand plant transcriptomes and the phylogenomics of green plants. Nature 574: 679–685. 31645766 10.1038/s41586-019-1693-2 PMC6872490

[ajb216108-bib-0084] Parham, J. F. , P. C. J. Donoghue , C. J. Bell , T. D. Calway , J. J. Head , P. A. Holroyd , J. G. Inoue , et al. 2012. Best practices for justifying fossil calibrations. Systematic Biology 61: 346–359. 22105867 10.1093/sysbio/syr107 PMC3280042

[ajb216108-bib-0085] Pereira, J. B. S. , A. M. Giulietti , J. Prado , S. Vasconcelos , M. T. C. Watanabe , D. S. B. Pinangé , R. R. M. Oliveira , et al. 2021. Plastome‐based phylogenomics elucidate relationships in rare *Isoetes* species groups from the Neotropics. Molecular Phylogenetics and Evolution 161: 107177. 33866010 10.1016/j.ympev.2021.107177

[ajb216108-bib-0086] Pereira, J. B. S. , P. H. Labiak , T. Stützel , and C. Schulz . 2017. Origin and biogeography of the ancient genus *Isoetes* with focus on the Neotropics. Botanical Journal of the Linnean Society 185: 253–271.

[ajb216108-bib-0087] Pfeiffer, N. E. 1922. Monograph of the Isoetaceae. Annals of the Missouri Botanical Garden 9: 79–232.

[ajb216108-bib-0088] Pierce, R. L. 1961. Lower Upper Cretaceous plant microfossils from Minnesota. Minnesota Geological Survey Bulletin 42: 1–86.

[ajb216108-bib-0089] Pigg, K. B. 1992. Evolution of isoetalean lycopsids. Annals of the Missouri Botanical Garden 79: 589–612.

[ajb216108-bib-0090] Pigg, K. B. 2001. Isoetalean lycopsid evolution: from the Devonian to the present. American Fern Journal 91: 99–114.

[ajb216108-bib-0091] Pollock, D. D. , D. J. Zwickl , J. A. McGuire , and D. M. Hillis . 2002. Increased taxon sampling is advantageous for phylogenetic inference. Systematic Biology 51: 664–671. 12228008 10.1080/10635150290102357 PMC2943957

[ajb216108-bib-0092] PPG I . 2016. A community‐derived classification for extant lycophytes and ferns. Journal of Systematics and Evolution 54: 563–603.

[ajb216108-bib-0093] Prestianni, C. , and R. W. Gess . 2014. The rooting system of *Leptophloeum* Dawson: New material from the Upper Devonian, Famennian Witpoort Formation of South Africa. Review of Palaeobotany and Palynology 209: 35–40.

[ajb216108-bib-0094] Rambaut, A. , A. J. Drummond , D. Xie , G. Baele , and M. A. Suchard . 2018. Posterior summarization in Bayesian phylogenetics using Tracer 1.7. Systematic Biology 67: 901–904. 29718447 10.1093/sysbio/syy032 PMC6101584

[ajb216108-bib-0095] Renzaglia, K. S. , B. Crandall‐Stotler , S. Pressel , J. G. Duckett , S. Schuette , and P. K. Strother . 2015. Permanent spore dyads are not ‘a thing of the past’: on their occurrence in the liverwort *Haplomitrium* (Haplomitriopsida). Botanical Journal of the Linnean Society 179: 658–669.

[ajb216108-bib-0096] Retallack, G. J. 1997. Earliest Triassic origin of *Isoetes* and quillwort evolutionary radiation. Journal of Paleontology 71: 500–521.

[ajb216108-bib-0097] Ronquist, F. , and J. P. Huelsenbeck . 2003. MRBAYES 3: Bayesian phylogenetic inference under mixed models. Bioinformatics 19: 1572–1574. 12912839 10.1093/bioinformatics/btg180

[ajb216108-bib-0098] Rosenberg, M. S. , and S. Kumar . 2001. Incomplete taxon sampling is not a problem for phylogenetic inference. Proceedings of the National Academy of Sciences, USA 98: 10751–10756. 10.1073/pnas.191248498 PMC58547 11526218

[ajb216108-bib-0099] Rubinstein, C. V. , P. Gerrienne , G. S. de la Puente , R. A. Astini , and P. Steemans . 2010. Early Middle Ordovician evidence for land plants in Argentina (eastern Gondwana). New Phytologist 188: 365–369. 20731783 10.1111/j.1469-8137.2010.03433.x

[ajb216108-bib-0100] Rydin, C. , R. Blokzijl , O. Thureborn , and N. Wikström . 2021. Node ages, relationships, and phylogenomic incongruence in an ancient gymnosperm lineage – phylogeny of *Ephedra* revisited. Taxon 70: 701–719.

[ajb216108-bib-0101] Rydin, C. , and M. Källersjö . 2002. Taxon sampling and seed plant phylogeny. Cladistics 18: 485–513. 34911214 10.1111/j.1096-0031.2002.tb00288.x

[ajb216108-bib-0102] Rydin, C. , and N. Wikström . 2002. Phylogeny of *Isoetes* (Lycopsida): resolving basal relationships using *rbcL* sequences. Taxon 51: 83–89.

[ajb216108-bib-0103] Sauquet, H. , S. Y. W. Ho , M. A. Gandolfo , G. J. Jordan , P. Wilf , D. J. Cantrill , M. J. Bayly , et al. 2012. Testing the impact of calibration on molecular divergence times using a fossil‐rich group: the case of *Nothofagus* (Fagales). Systematic Biology 61: 289–313. 22201158 10.1093/sysbio/syr116

[ajb216108-bib-0104] Schmidt, A. R. , L. Regalado , S. Weststrand , P. Korall , E.‐M. Sadowski , H. Schneider , E. Jansen , et al. 2020. *Selaginella* was hyperdiverse already in the Cretaceous. New Phytologist 228: 1176–1182. 32282937 10.1111/nph.16600

[ajb216108-bib-0105] Schuettpelz, E. , and S. B. Hoot . 2006. Inferring the root of *Isoëtes*: exploring alternatives in the absence of an acceptable outgroup. Systematic Botany 31: 258–270.

[ajb216108-bib-0106] Schulte, J. A. 2013. Undersampling taxa will underestimate molecular divergence dates: an example from the South American lizard clade Liolaemini. International Journal of Evolutionary Biology 2013: 628467. 24222886 10.1155/2013/628467 PMC3809987

[ajb216108-bib-0107] Seton, M. , R. D. Müller , S. Zahirovic , C. Gaina , T. Torsvik , G. Shephard , A. Talsma , et al. 2012. Global continental and ocean basin reconstructions since 200 Ma. Earth‐Science Reviews 113: 212–270.

[ajb216108-bib-0108] Simpson, J. T. , K. Wong , S. D. Jackman , J. E. Schein , S. J. M. Jones , and I. Birol . 2009. ABySS: a parallel assembler for short read sequence data. Genome Research 19: 1117–1123. 19251739 10.1101/gr.089532.108 PMC2694472

[ajb216108-bib-0109] Skog, J. E. , and C. R. Hill . 1992. The Mesozoic herbaceous lycopsids. Annals of the Missouri Botanical Garden 79: 648–675.

[ajb216108-bib-0110] Srivastava, B. 1946. Silicified plant‐remains from the Rajmahal series of India. Proceedings of the National Academy of Sciences, India 15: 185–211.

[ajb216108-bib-0111] Staden, R. 1996. The Staden sequence analysis package. Molecular Biotechnology 5: 233–241. 8837029 10.1007/BF02900361

[ajb216108-bib-0112] Staden, R. , K. F. Beal , and J. K. Bonfield . 2000. The Staden package, 1998. *In* S. Misener and S. A. Krawets [eds.], Bioinformatics methods and protocols, Methods in Molecular Biology, 132, 115–130. Humana Press, NJ, USA. 10.1385/1-59259-192-2:115 10547834

[ajb216108-bib-0113] Straub, S. C. K. , M. Fishbein , T. Livshultz , Z. Foster , M. Parks , K. Weitemier , R. C. Cronn , et al. 2011. Building a model: developing genomic resources for common milkweed (*Asclepias syriaca*) with low coverage genome sequencing. BMC Genomics 12: 211. 21542930 10.1186/1471-2164-12-211 PMC3116503

[ajb216108-bib-0114] Straub, S. C. K. , M. Parks , K. Weitemier , M. Fishbein , R. C. Cronn , and A. Liston . 2012. Navigating the tip of the genomic iceberg: next‐generation sequencing for plant systematics. American Journal of Botany 99: 349–364. 22174336 10.3732/ajb.1100335

[ajb216108-bib-0115] Strother, P. K. , S. Al‐Hajri , and A. Traverse . 1996. New evidence for land plants from the lower Middle Ordovician of Saudi Arabia. Geology 24: 55–58.

[ajb216108-bib-0116] Su, D. , L. Yang , X. Shi , X. Ma , X. Zhou , S. B. Hedges , and B. Zhong . 2021. Large‐scale phylogenomic analyses reveal the monophyly of bryophytes and Neoproterozoic origin of land plants. Molecular Biology and Evolution 38: 3332–3344. 33871608 10.1093/molbev/msab106 PMC8321542

[ajb216108-bib-0117] Sullivan, A. R. , B. Schiffthaler , S. L. Thompson , N. R. Street , and X.‐R. Wang . 2017. Interspecific plastome recombination reflects ancient reticulate evolution in *Picea* (Pinaceae). Molecular Biology and Evolution 34: 1689–1701. 28383641 10.1093/molbev/msx111 PMC5455968

[ajb216108-bib-0118] Taylor, T. N. , E. L. Taylor , and M. Krings . 2009. Paleobotany: the biology and evolution of fossil plants. Academic Press, NY, NY, USA.

[ajb216108-bib-0119] Taylor, W. C. , and R. J. Hickey . 1992. Habitat, evolution and speciation in *Isoetes* . Annals of the Missouri Botanical Garden 79: 613–622.

[ajb216108-bib-0120] Testo, W. , A. Field , and D. Barrington . 2018. Overcoming among‐lineage rate heterogeneity to infer the divergence times and biogeography of the clubmoss family Lycopodiaceae. Journal of Biogeography 45: 1929–1941.

[ajb216108-bib-0121] Thomas, B. A. 1992. Paleozoic herbaceous lycopsids and the beginnings of extant *Lycopodium* sens. lat. and *Selaginella* sens. lat. Annals of the Missouri Botanical Garden 79: 623–631.

[ajb216108-bib-0122] Thomas, B. A. 1997. Upper Carboniferous herbaceous lycopsids. Review of Palaeobotany and Palynology 95: 129–153.

[ajb216108-bib-0123] Thorne, J. L. , and H. Kishino . 2002. Divergence time and evolutionary rate estimation with multilocus data. Systematic Biology 51: 689–702. 12396584 10.1080/10635150290102456

[ajb216108-bib-0124] Troìa, A. , J. B. Pereira , C. Kim , and W. C. Taylor . 2016. The genus *Isoetes* (Isoetaceae): a provisional checklist of the accepted and unresolved taxa. Phytotaxa 277: 101–145.

[ajb216108-bib-0125] Walker, J. D. , J. W. Geissman , S. A. Bowring , and L. E. Babcock . 2018. Geologic time scale v. 5.0. Available at 10.1130/2018.CTS005R3C. Geological Society of America, Boulder, CO, USA.

[ajb216108-bib-0126] Wang, Y. , and C. M. Berry . 2003. A novel lycopsid from the Upper Devonian of Jiangsu, China. Palaeontology 46: 1297–1311.

[ajb216108-bib-0127] Wellman, C. H. 2010. The invasion of the land by plants: when and where? New Phytologist 188: 306–309. 20941845 10.1111/j.1469-8137.2010.03471.x

[ajb216108-bib-0128] Wellman, C. H. , P. G. Gensel , and W. A. Taylor . 2009. Spore wall ultrastructure in the early lycopsid *Leclercqia* (Protolepidodendrales) from the Lower Devonian of North America: evidence for a fundamental division in the lycopsids. American Journal of Botany 96: 1849–1860. 21622306 10.3732/ajb.0800422

[ajb216108-bib-0129] Wellman, C. H. , P. L. Osterloff , and U. Mohiuddin . 2003. Fragments of the earliest land plants. Nature 425: 282–285. 13679913 10.1038/nature01884

[ajb216108-bib-0130] Weststrand, S. , and P. Korall . 2016. Phylogeny of Selaginellaceae: There is value in morphology after all! American Journal of Botany 103: 2136–2159. 27999082 10.3732/ajb.1600156

[ajb216108-bib-0131] Wikström, N. 2001. Diversification and relationships of extant homosporous lycopods. American Fern Journal 91: 150–165, 16.

[ajb216108-bib-0132] Wikström, N. , B. Bremer , and C. Rydin . 2020. Conflicting phylogenetic signals in genomic data of the coffee family (Rubiaceae). Journal of Systematics and Evolution 58: 440–460.

[ajb216108-bib-0133] Wikström, N. , and P. Kenrick . 1997. Phylogeny of Lycopodiaceae (Lycopsida) and the relationship of *Phylloglossum drumondii* Kunze based on *rbcL* sequence data. International Journal of Plant Sciences 158: 862–871.

[ajb216108-bib-0134] Wikström, N. , and P. Kenrick . 2000. Relationships of *Lycopodium* and *Lycopodiella* based on combined plastid *rbcL* gene and *trnL* intron sequence data. Systematic Botany 25: 495–510, 16.

[ajb216108-bib-0135] Wikström, N. , P. Kenrick , and M. Chase . 1999. Epiphytism and terrestrialization in tropical *Huperzia* (Lycopodiaceae). Plant Systematics and Evolution 218: 221–243.

[ajb216108-bib-0136] Wilce, J. H. 1972. Lycopod spores, I. General spore patterns and the generic segregates of *Lycopodium* . American Fern Journal 62: 65–79.

[ajb216108-bib-0137] Wood, D. , G. Besnard , D. J. Beerling , C. P. Osborne , and P. A. Christin . 2020. Phylogenomics indicates the “living fossil” *Isoetes* diversified in the Cenozoic. PLoS One 15: e0227525. 32555586 10.1371/journal.pone.0227525 PMC7302493

[ajb216108-bib-0138] Worobey, M. , G.‐Z. Han , and A. Rambaut . 2014. A synchronized global sweep of the internal genes of modern avian influenza virus. Nature 508: 254–257. 24531761 10.1038/nature13016 PMC4098125

[ajb216108-bib-0139] Zhang, H.‐R. , R. Wei , Q.‐P. Xiang , and X.‐C. Zhang . 2020. Plastome‐based phylogenomics resolves the placement of the sanguinolenta group in the spikemoss of lycophyte (Selaginellaceae). Molecular Phylogenetics and Evolution 147: 106788. 32173413 10.1016/j.ympev.2020.106788

[ajb216108-bib-0140] Zhou, X.‐M. , C. J. Rothfels , L. Zhang , Z.‐R. He , T. Le Péchon , H. He , N. T. Lu , et al. 2016. A large‐scale phylogeny of the lycophyte genus *Selaginella* (Selaginellaceae: Lycopodiopsida) based on plastid and nuclear loci. Cladistics 32: 360–389. 34740298 10.1111/cla.12136

[ajb216108-bib-0141] Zwickl, D. J. , and D. M. Hillis . 2002. Increased taxon sampling greatly reduces phylogenetic error. Systematic Biology 51: 588–598. 12228001 10.1080/10635150290102339

