# Supplementary material for: No phylogenomic support for a Cenozoic origin of the “living fossil” Isoetes
Source: Am J Bot. 2023 Jan 2;110(1):e16108. doi: 10.1002/ajb2.16108 (PMC10108322; doi:10.1002/ajb2.16108)
Supplement: Supplementary file 1 — Appendix S1. List of lab identification numbers, taxon names, taxon distribution, DNA voucher information and GenBank accession numbers for sequences used in the study. [file AJB2-110-0-s001.pdf]

**Appendix S1.** List of lab identification numbers, taxon names, taxon distribution, DNA voucher information (including area and year of collection ) for newly produced data, and accession numbers for sequences used in the analyses. References are given for sequences taken from GenBank. Ribosomal DNA cistron sequences were extracted from transcriptome sequences published by the 1000 plant (1KP) initiative[1]. Taxon distributions were extracted from GBIF[2]. Area names follow the World Geographical Scheme for Recording Plant Distributions[3] except the Mediterranean distribution, which refers to an occurrence in either one of the 22 sovereign countries in Europe, Africa and Temperate Asia that borders the Mediterranean Sea.

| Lab. ident. | Taxon | Distribution | DNA voucher | Area and year of collection | Plastid accession number | rDNA cistron accession number |
|---|---|---|---|---|---|---|
| EL002 | *Isoetes aequinoctialis* Welw. ex A.Braun | Southern and Trop. Africa | Kornas 3453 (BR) | Zambia 1973 | ON017796 | ON010717 |
| EL026 | *Isoetes azorica* Durieu ex Milde | The Azores | Gonalves 2611 (BM) | Azores 1971 | ON060511 | ON010729 |
| EL017 | *Isoetes australis* S.Williams | Western Australia | Orchard 1274 (MEL) | Australia 1968 | ON060521 | ON010724 |
| EL030 | *Isoetes biafrana* Alston | Trop. Africa | Le Testu 3016 (BM) | C. Afr. Rep. 1951 | ON060504 | ON010733 |
| EL012 | *Isoetes cubana* Engelm. | Caribbean; Central America; Mexico | Hickey & Hickey 979 (W) | Mexico 1986 | ON060519 | ON010721 |
| EL022 | *Isoetes drummondii* A.Braun | Australia | Beauglehole 75018 (MEL) | Australia 1983 | ON060513 | ON010727 |
| EL027 | *Isoetes durieui* Bory | Mediterranean; SW Europe | Byfield s.n. (BM) | Turkey 1992 | ON060505 | ON010730 |
| EL028 | *Isoetes durieui* Bory | Mediterranean; SW Europe | De Retz 65141 (BM) | France 1972 | ON060506 | ON010731 |
| EL013 | *Isoetes echinospora* Durieu | Europe; N. America; Asia-Temperate | Ford 609 (W) | Canada 2006 | ON060510 | ON010722 |
| | *Isoetes flaccida* Shuttlew. ex A.Braun | Southeastern USA | --- | --- | NC_014675[4] | --- |
| EL005 | *Isoetes giessii* Launert | Namibia | Giess, Volk & Bleissner 5564 (BR) | Namibia 1963 | ON060502 | ON010718 |
| EL025 | *Isoetes lechleri* Mett. | Western S. America | Fernandez-Casas & Molero 6619 (BM) | Bolivia 1982 | ON060507 | ON010728 |
| EL032 | *Isoetes malinverniana* Ces & De Not. | Italy | Raynal 20885 (BR) | Italy 1978 | ON060522 | ON010734 |
| EL020 | *Isoetes neoguineensis* Baker | Papua New Guinea | Craven 2717 (MEL) | Papua New Guinea 1974 | ON060515 | ON010725 |
| EL008 | *Isoetes nigritiana* A.Braun | W. Trop. Africa; West-Central Trop. Africa | De Wilde & De Wilde-Duyfjes 3518 (BR) | Cameroon 1964 | ON060516 | ON010719 |
| EL033 | *Isoetes occidentalis* L.F.Hend. | Subarctic America; Western Canada; Northwestern USA; Southwestern USA | Oettinger & Thorne 1268 (BM) | USA 1969 | ON060509 | ON010735 |
| EL015 | *Isoetes pallida* Hickey | Mexico | Hickey & Hickey 962 (W) | Mexico 1986 | ON060520 | ON010723 |
| EL036 | *Isoetes philippinensis* Merr. & L.M.Perry | Malesia | Price 500 (BM) | Philippines 1969 | ON060524--ON060589 | ON010737 |
| EL021 | *Isoetes pusilla* C.R.Marsden & Chinnock | New South Wales; Victoria | Willis s.n. (MEL) | Australia 1981 | ON060512 | ON010726 |
| EL039 | *Isoetes sampathkumaranii* L.N.Rao | Indian Subcontinent | Goswami s.n. (BM) | India (unknown year) | ON060514 | ON010739 |
| EL035 | *Isoetes schweinfurthii* A.Braun | Trop. Africa | Kers 3130 (BM) | Namibia 1968 | ON060518 | ON010736 |
| EL037 | *Isoetes transvaalensis* C.Jermy & Schelpe | Southern Africa | Hilliard & Burtt 5989 (BM) | South Africa 1969 | ON060503 | ON010738 |
| EL010 | *Isoetes weberi* Herter | Brazil | Callé 95840 (BR) | Brazil 1935 | ON060508 | ON010720 |
| EL029 | *Isoetes welwitschii* A.Braun | Trop. Africa | Wingfield 2032 (BM) | Tanzania 1972 | ON060517 | ON010732 |
| EL057 | *Isoetes wormaldii* Sim. | South Africa | Pocock 20009 (BM) | South Africa 1955 | ON060523 | ON010740 |
| | *Huperzia lucidula* (Michx.) Trevis | --- | --- | --- | NC_006861[5] | |
| | ~ | --- | --- | --- | --- | GKAG[1] |
| | *Lycopodium clavatum* L. | --- | --- | --- | MH549642[6] | --- |

| Lab. ident. | Taxon | Distribution | DNA voucher | Area and year of collection | Plastid accession number | rDNA cistron accession number |
|---|---|---|---|---|---|---|
| | *Lycopodium annotinum* L. | --- | --- | --- | --- | ENQF[1] |
| | *Selaginella kraussiana* (Kunze) A.Braun | --- | --- | --- | NC_040926[6] | --- |
| | *Selaginella moellendorffii* Hieron. | --- | --- | --- | HM173080[7] | |
| | ~ | --- | --- | --- | --- | ZZOL[1] |
| | *Selaginella selaginoides* (L.) P.Beauv. ex Schrank & Mart. | --- | --- | --- | --- | KUXM[1] |
| | *Aneura mirabilis* (Malmb.) Wickett & Goffinet | --- | --- | --- | NC_010359[8] | |
| | *Pellia neesiana* (Gottsche) Limpr. | --- | --- | --- | --- | JHFI[1] |
| | *Marchantia paleacea* Bertol. | --- | --- | --- | NC_001319[9] | |
| | ~ | --- | --- | --- | --- | HMHL[1] |
| | *Physcomitrella patens* (Hedw.) Bruch & Schimp. | --- | --- | --- | AP005672[10] | |
| | *Physcomitrium* sp. | --- | --- | --- | --- | YEPO[1] |
| | *Syntrichia ruralis* (Hedw.) F.Weber & D.Mohr | --- | --- | --- | FJ546412[11] | |
| | *Syntrichia princeps* (De Not.) Mitt. | --- | --- | --- | --- | GRKU[1] |
| | *Anthoceros angustus* Steph. | --- | --- | --- | NC_004543[12] | |
| | *Anthoceros agrestis* Paton | --- | --- | --- | --- | BSNI[1] |
| | *Diplopterygium glaucum* (Thunb. ex Houtt.) Nakai | --- | --- | --- | NC_024158[13] | |
| | *Hymenophyllum cupressiforme* Labill. | --- | --- | --- | --- | TRPJ[1] |
| | *Pteridium aquilinum* (L.) Kuhn | --- | --- | --- | NC_014348[14] | |
| | *Lygodium japonicum* (Thunb.) Sw. | --- | --- | --- | KF225593[13] | |
| | ~ | --- | --- | --- | --- | PBUU[1] |
| | *Osmundastrum cinnamomeum* (L.) C.Presl | --- | --- | --- | NC_024157[13] | |
| | ~ | --- | --- | --- | --- | RFMZ[1] |
| | *Ginkgo biloba* L. | --- | --- | --- | NC_016986[15] | |
| | ~ | --- | --- | --- | --- | SGTW[1] |
| | *Welwitschia mirabilis* Hook.f. | --- | --- | --- | NC_010654[16] | |
| | *Gnetum montanum* Markgr. | --- | --- | --- | --- | GTHK[1] |
| | *Pinus thunbergii* Parl. | --- | --- | --- | D17510[17] | |
| | *Pinus radiata* D.Don | --- | --- | --- | --- | DZQM[1] |
| | *Amborella trichopoda* Baill. | --- | --- | --- | NC_005086[18] | |
| | ~ | --- | --- | --- | --- | URDJ[1] |
| | *Nymphaea alba* L. | --- | --- | --- | AJ627251[19] | |
| | *Nymphaea* sp. | --- | --- | --- | --- | PZRT[1] |
| | *Nuphar advena* (Aiton) W.T.Aiton | --- | --- | --- | DQ354691[20] | |
| | ~ | --- | --- | --- | --- | WTKZ[1] |

| Lab. ident. | Taxon | Distribution | DNA voucher | Area and year of collection | Plastid accession number | rDNA cistron accession number |
|---|---|---|---|---|---|---|
| | *Ananas comosus* (L.) Merr. | --- | --- | --- | NC_026220[21] | |
| | *Typha latifolia* L. | --- | --- | --- | --- | BRUD[1] |
| | *Ceratophyllum demersum* L. | --- | --- | --- | EF614270[22] | |
| | ~ | --- | --- | --- | --- | NPND[1] |
| | *Ranunculus macranthus* Scheele | --- | --- | --- | NC_008796[20] | |
| | *Hydrastis canadensis* L. | --- | --- | --- | --- | VGHH[1] |
| | *Sesamum indicum* L. | --- | --- | --- | JN637766[23] | |
| | *Amelanchier canadensis* (L.) Medik. | --- | --- | --- | --- | EAVM[1] |
| | *Veronica nakaiana* Ohwi | --- | --- | --- | NC_031153[24] | |
| | *Synphoricarpos* sp. | --- | --- | --- | --- | CAQZ[1] |
| | *Plantago maritima* L. | --- | --- | --- | NC_028519[25] | |
| | ~ | --- | --- | --- | --- | YKZB[1] |

## References


[1]One Thousand Plant Transcriptomes Initiative 2019. One thousand plant transcriptomes and the phylogenomics of green plants. *Nature* 574: 679–685.

[2]GBIF.org 2021. GBIF Home Page. Available from: https://www.gbif.org [accessed January 29th, 2021].

[3]Brummit, R. K. 2001. *World geographic scheme for recording plant distributions*, 2nd edn. Hunt Institute for Botanical Documentation, Carnegie Mellon University, Pittsburgh.

[4]Karol, K. G., K. Arumuganathan, J. L. Boore, A. M. Duffy, K. D. Everett, J. D. Hall, S. K. Hansen, et al. 2010. Complete plastome sequences of *Equisetum arvense* and *Isoetes flaccida*: implications for phylogeny and plastid genome evolution of early land plant lineages, *BMC Evolutionary Biology* 10: 321.

[5]Wolf, P. G, K. G. Karol, D. F. Mandoli, J. Kuehl, K. Arumuganathan, M. W. Ellis, B. D. Mishler, et al. 2005. The first complete chloroplast genome sequence of a lycophyte, *Huperzia lucidula* (Lycopodiaceae), *Gene* 350(2): 117–128.

[6]Mower, J. P., P. F. Ma, F. Grewe, A. Taylor, T. P. Michael, R. VanBuren, and Y.L. Qiu, Y.L. 2019. Lycophyte plastid genomics: extreme variation in GC, gene and intron content and multiple inversions between a direct and inverted orientation of the rRNA repeat. *New Phytologist* 222: 1061–1075.

[7]Liu, Y., B. Wang, P. Cui, L. Li, J. Y. Xue, J. Yu, and Y. L. Qiu. 2012. The mitochondrial genome of the lycophyte *Huperzia squarrosa*: The most archaic form in vascular plants. *PLoS ONE* 7: E35168.

[8]Wickett, N. J., Y. Zhang, S. K. Hansen, J. M. Roper, J. V. Kuehl, S. A. Plock, P. G. Wolf, et al. 2008. Functional gene losses occur with minimal size reduction in the plastid genome of the parasitic liverwort *Aneura mirabilis*. *Molecular Biology and Evolution* 25: 393–401.

[9]Umesono, K., H. Inokuchi, K. Ohyama, and H. Ozeki. 1984. Nucleotide sequence of *Marchantia polymorpha* chloroplast DNA: a region possibly encoding three tRNAs and three proteins including a homologue of *E. coli* ribosomal protein S14. *Nucleic Acids Research* 12: 9551–9565.

[10]Sugiura, C., Y. Kobayashi, S. Aoki, C. Sugita, and M. Sugita. 2003. Complete chloroplast DNA sequence of the moss *Physcomitrella patens*: evidence for the loss and relocation of *rpoA* from the chloroplast to the nucleus. *Nucleic Acids Research* 31: 5324–5331.

[11]Oliver, M. J., A. G. Murdock, B. D. Mishler, J. V. Kuehl, J. L. Boore, D. F. Mandoli, K. D. Everett, et al. 2010. Chloroplast genome sequence of the moss *Tortula ruralis:* gene content, polymorphism, and structural arrangement relative to other green plant chloroplast genomes. *BMC Genomics* 11: 143.

[12]Kugita, M., A. Kaneko, Y. Yamamoto, Y. Takeya, T. Matsumoto, and K. Yoshinaga. 2003. The complete nucleotide sequence of the hornwort (*Anthoceros formosae*) chloroplast genome: insight into the earliest land plants. *Nucleic Acids Research* 31: 716–721.

[13]Kim, H. T., M. G. Chung, and K. J. Kim. 2014. Chloroplast genome evolution in early diverged leptosporangiate ferns. *Molecular Cell* 37: 372–382.

[14]Der, J. P., A. M. Duffy, M. Kusner, C. Gu, P. Overvoorde, and P. G. Wolf. 2010. Direct submission, 27 July 2010.

[15]Li, X., Q. Li, X. Lin, Z. Hu, and S. Chen. 2012. Direct submission, 15 March 2012.

[16]McCoy, S. R., J. V. Kuehl, J. L. Boore, and L. A. Raubeson. 2008. The complete plastid genome sequence of *Welwitschia mirabilis*: an unusually compact plastome with accelerated divergence rates. *BMC Evolutionary Biology* 8: 130.

[17]Wakasugi, T., J. Tsudzuki, S. Ito, K. Nakashima, T. Tsudzuki, and M. Sugiura. 1994. Loss of all *ndh* genes as determined by sequencing the entire chloroplast genome of the black pine *Pinus thunbergii*. *Proceedings of the National Academy of Sciences, U.S.A.* 91: 9794–9798.

[18]Goremykin, V. V., K. I. Hirsch-Ernst, S. Wolfl, and F. H. Hellwig. 2003. Analysis of the *Amborella trichopoda* chloroplast genome sequence suggests that *Amborella* is not a basal angiosperm. *Molecular Biology and Evolution* 20: 1499–1505.

[19]Goremykin, V. V., K. I. Hirsch-Ernst, S. Wolfl, and F. H. Hellwig. 2004. The chloroplast genome of *Nymphaea alba*: whole-genome analyses and the problem of identifying the most basal angiosperm. *Molecular Biology and Evolution* 21: 1445–1454.

[20]Raubeson, L. A., R. Peery, T. Chumley, C. Dziubek, M. Fourcade, J. Boore, and R. Jansen. 2007. Comparative chloroplast genomics: analyses including new sequences from the angiosperms *Nuphar advena* and *Ranunculus macranthus*. *BMC Genomics* 8: 174.

[21]Nashima, K., S. Terakami, C. Nishitani, M. Kunihisa, M. Shoda, M. Takeuchi, N. Urasaki, et al. 2015. Complete chloroplast genome sequence of pineapple (*Ananas comosus*). *Tree Genetics and Genomes* 11: 60.

[22]Moore, M. J., C. D. Bell, P. S. Soltis, and D. E. Soltis. 2007. Using plastid genome-scale data to resolve enigmatic relationships among basal angiosperms. *Proceedings of the National Academy of Sciences, U.S.A.* 104: 19363–19368.

[23]Yi, D. K., and K. J. Kim. 2012. Complete chloroplast genome sequences of important oilseed crop *Sesamum indicum* L. *PLoS ONE* 7: E35872.

[24]Choi, K. S., M. G. Chung, and S. Park. 2016. The complete chloroplast genome sequences of three Veroniceae species (Plantaginaceae): comparative analysis and highly divergent regions. *Frontiers in Plant Science* 7: 355.

[25]Zhu, A., W. Guo, S. Gupta, W. Fan, and J. P. Mower. 2016. Evolutionary dynamics of the plastid inverted repeat: the effects of expansion, contraction, and loss on substitution rates. *New Phytologist* 209: 1747–1756.
