# Supplementary material for: No phylogenomic support for a Cenozoic origin of the “living fossil” Isoetes
Source: Am J Bot. 2023 Jan 2;110(1):e16108. doi: 10.1002/ajb2.16108 (PMC10108322; doi:10.1002/ajb2.16108)
Supplement: Supplementary file 2 — Appendix S2. Phylograms and chronograms resulting from non‐clock and relaxed‐clock analyses of plastid data and nuclear ribosomal cistron data. [file AJB2-110-0-s003.pdf]

Appendix S2. Phylograms and chronograms resulting from non-clock and relaxed-clock analyses of plastid data and nuclear ribosomal cistron data.

Figure S1 Phylogram resulting from non-clock analysis of complete plastid CDS data.

Figure S2 Phylogram resulting from non-clock analysis of nuclear rDNA gene data (18S, 5.8S, 26S).

Figure S3 Chronogram showing estimated absolute ages resulting from the relaxed-clock analysis of complete plastid CDS data using the independent lognormal model (ILN) and a birth-death tree prior.

Figure S4 Chronogram showing estimated absolute ages resulting from the relaxed-clock analysis of complete plastid CDS data using the white noise model (WN) and a birth-death tree prior.

Figure S5 Chronogram showing estimated absolute ages resulting from the relaxed-clock analysis of complete plastid CDS data using the brownian motion model described by Thorne and Kishino (TK02) and a birth-death tree prior.

Figure S6 Chronogram showing estimated absolute ages resulting from the relaxed-clock analysis of nuclear rDNA gene data using the independent lognormal model (ILN) and a birth-death tree prior.

Figure S7 Chronogram showing estimated absolute ages resulting from the relaxed-clock analysis of nuclear rDNA gene data using the white noise model (WN) and a birth-death tree prior.

Figure S8 Chronogram showing estimated absolute ages resulting from the relaxed-clock analysis of nuclear rDNA gene data using the Brownian motion model described by Thorne and Kishino (TK02) and a birth-death tree prior.

References

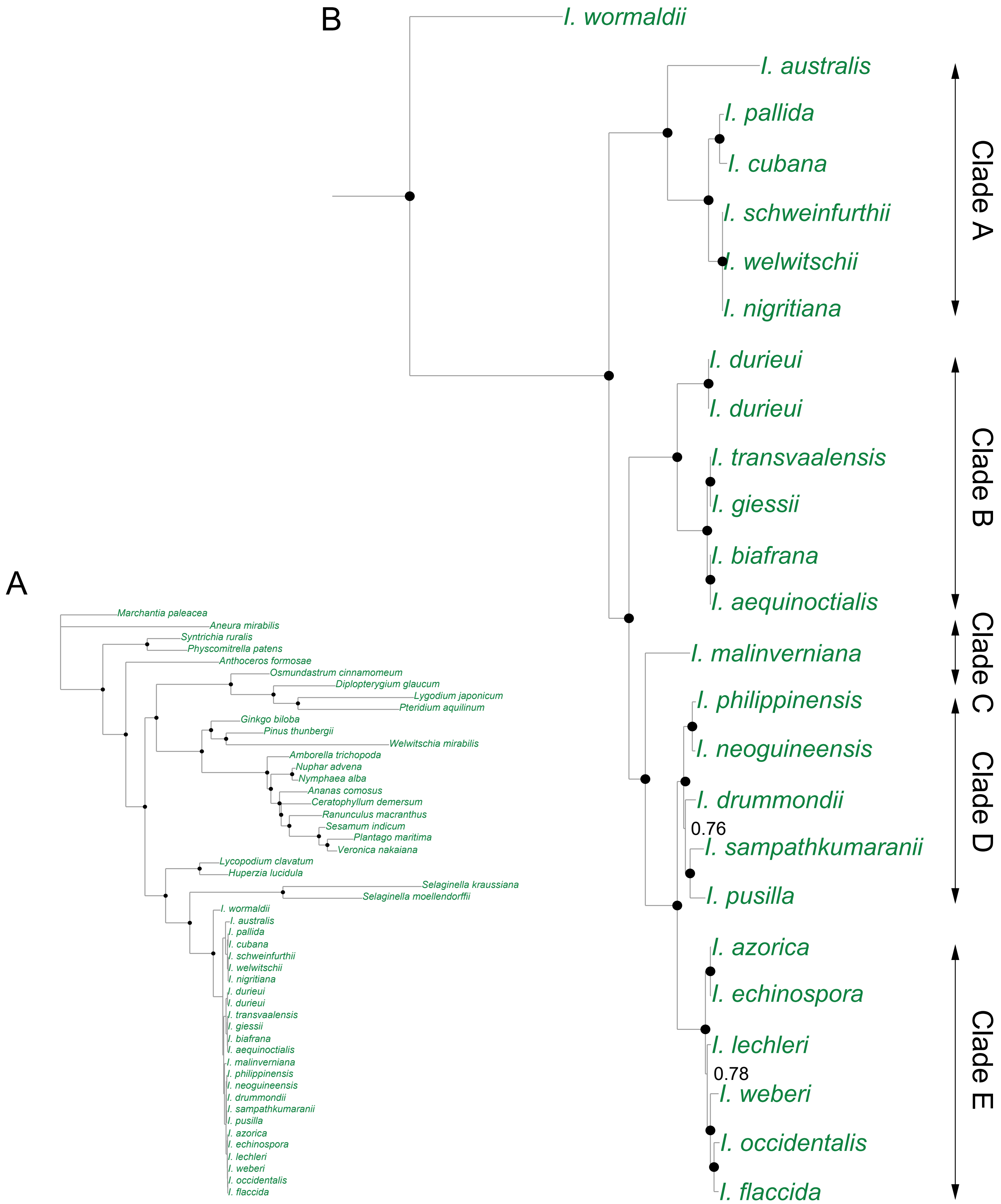

Figure S1 Phylogram resulting from non-clock analysis of complete plastid CDS data. The tree was rooted on *Marchantia paleaceae*. **(A)** Overview showing resolved relationships across the entire land plant clade. **(B)** Same tree focusing on the ingroup *Isoetes* with branch lengths upscaled more than 10 times. Nodes indicated by a black dot have a Bayesian posterior probability (BPP) of 1.00. Clades A to E sensu Larsén and Rydin (2016) are indicated to the right. *Isoetes wormaldii* (not included by Larsén and Rydin, 2016) was recently shown by Larsén et al. (2022) to be a divergent sister group to the remaining *Isoetes* and this is corroborated by the analysis of complete plastid CDS data presented here.

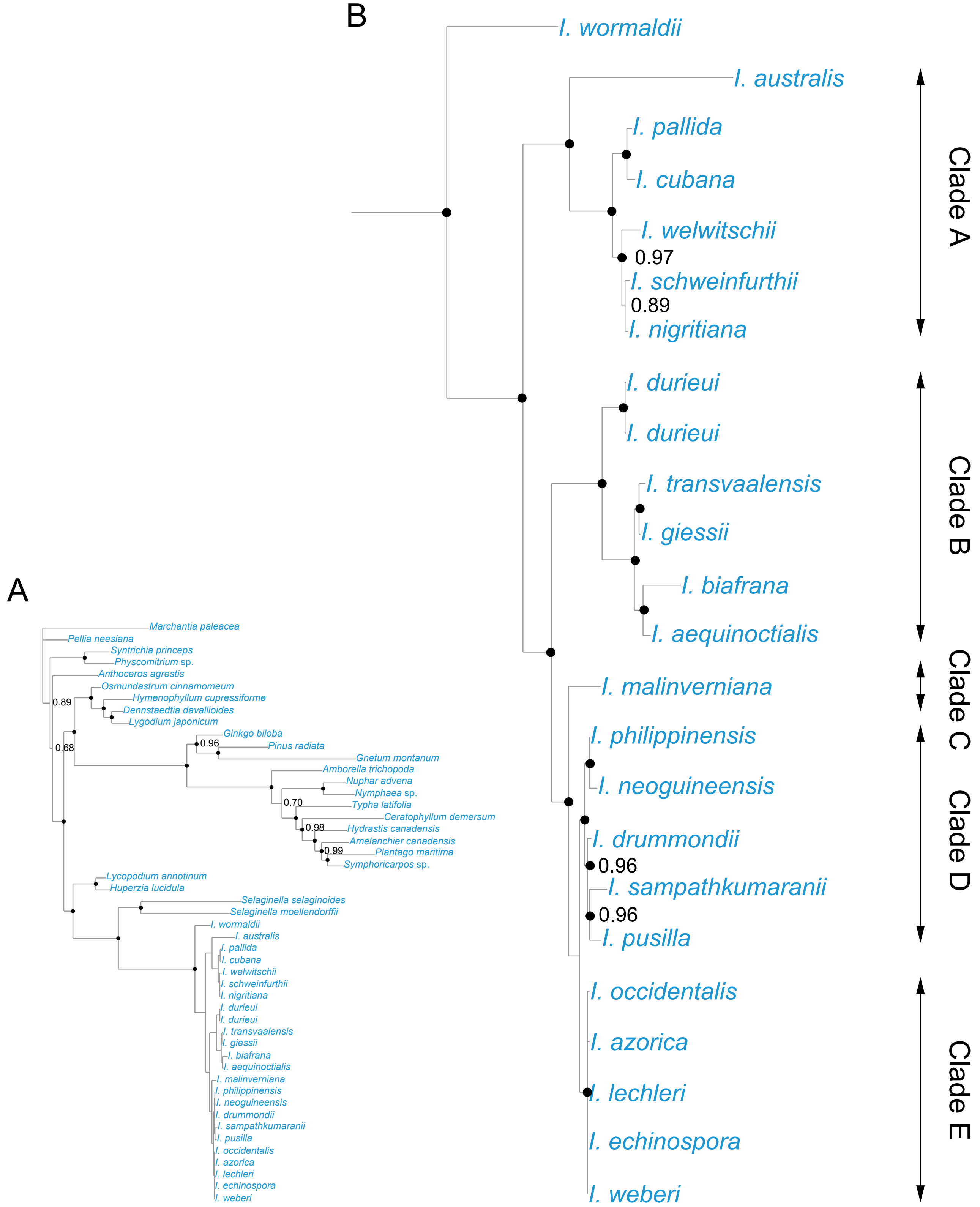

Figure S2 Phylogram resulting from non-clock analysis of nuclear rDNA gene data (18S, 5.8S, 26S). The tree was rooted on *Marchantia paleacea*. **(A)** Overview showing resolved relationships across the entire land plant clade. **(B)** Same tree focusing on the ingroup *Isoetes* with branch lengths upscaled more than 10 times. Nodes indicated by a black dot are well supported and have a Bayesian posterior probability (BPP) of 0.95 or more. Support values are 1.00 unless otherwise indicated in the figure. Clades A to E sensu Larsén and Rydin (2016) are indicated to the right. *Isoetes wormaldii* (not included in Larsén and Rydin, 2016) was recently shown by Larsén et al. (2022) to be a divergent sister group to the remaining *Isoetes* and this is corroborated by the analysis of nuclear ribosomal data presented here.

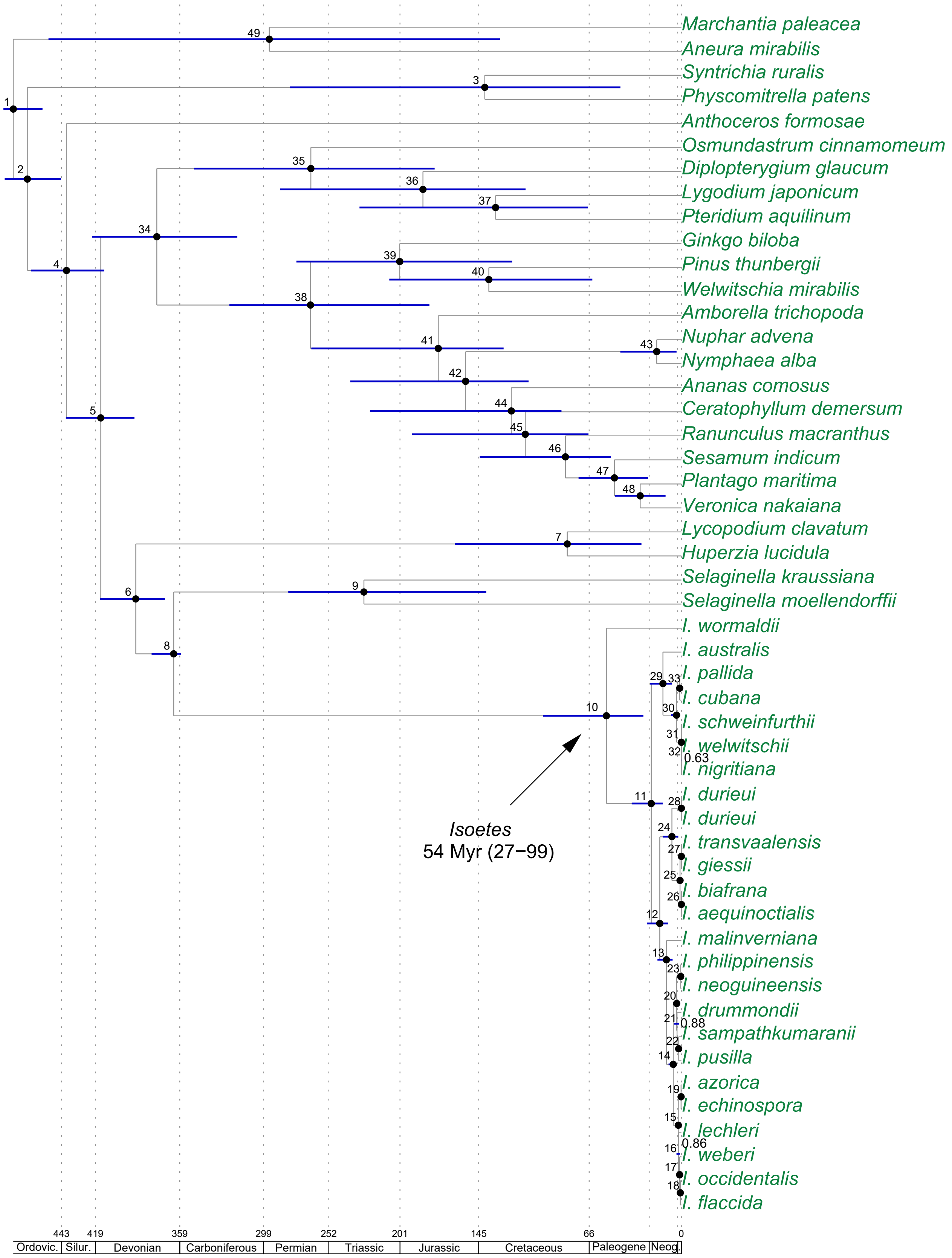

Figure S3 Chronogram showing estimated absolute ages resulting from the relaxed-clock analysis of complete plastid CDS data using the independent lognormal model (ILN) and a birth-death tree prior. Node heights are median ages. Confidence intervals (95% highest posterior density) of node ages are indicated as blue bars. Nodes indicated by a black dot have a Bayesian posterior probability (BPP) of 1.00. Detailed results for all nodes are reported in Appendix S3 and node numbers on the tree correspond to those used there. Estimated age for the *Isoetes* crown group is 54 Myr (27–99 Myr). Our node nr. 11 corresponds with the crown group *Isoetes* as estimated by Wood et al. (2020), and the estimated age 21 Myr (13–36 Myr) corresponds well with the age of 23 Myr (6–47 Myr) obtained in their analyses.

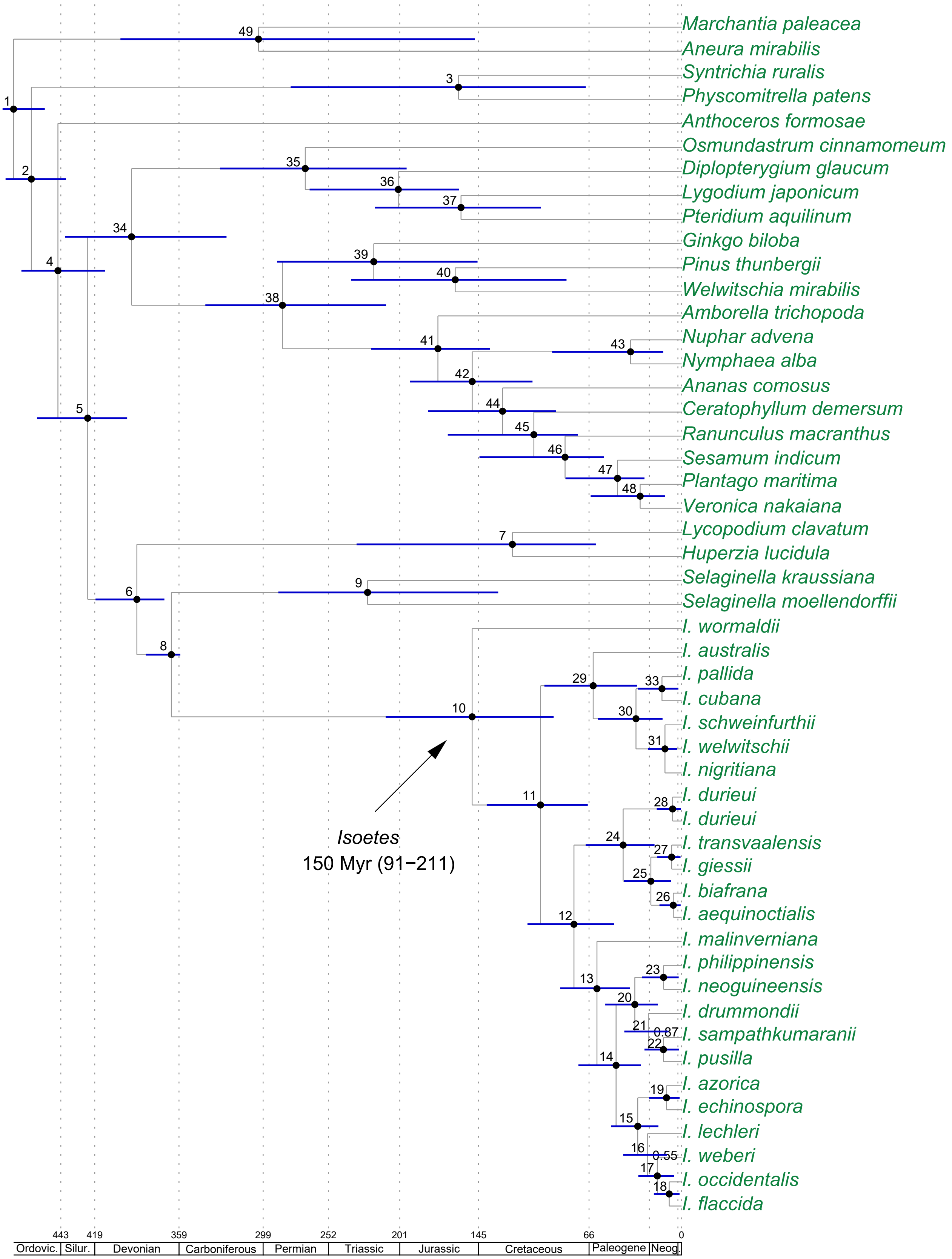

Figure S4 Chronogram showing estimated absolute ages resulting from the relaxed-clock analysis of complete plastid CDS data using the white noise model (WN) and a birth-death tree prior. Node heights are median ages. Confidence intervals (95% highest posterior density) of node ages are indicated as blue bars. Nodes indicated by a black dot have a Bayesian posterior probability (BPP) of 1.00. Detailed results for all nodes are reported in Appendix S3 and node numbers on the tree correspond to those used there. Estimated age for the *Isoetes* crown group is 150 Myr (91–211 Myr). Our node nr. 11 corresponds with the crown group *Isoetes* as estimated by Wood et al. (2020), and the estimated age 101 Myr (67–139 Myr) is considerably older than the age of 23 Myr (6–47 Myr) obtained in their analyses.

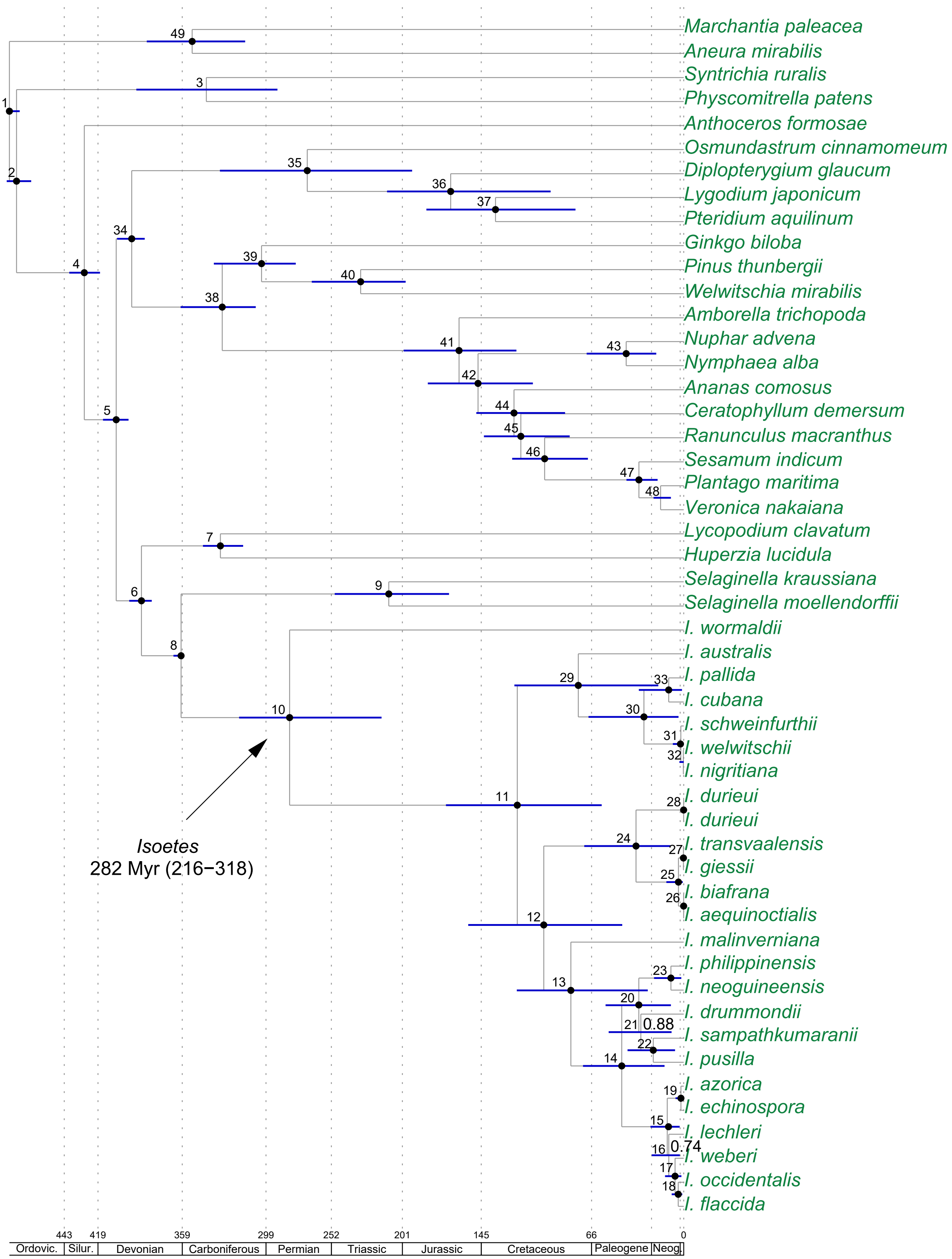

Figure S5 Chronogram showing estimated absolute ages resulting from relaxed-clock analysis of complete plastid CDS data using the brownian motion model described by Thorne and Kishino (TK02) and a birth-death tree prior. Node heights are median ages. Confidence intervals (95% highest posterior density) of node ages are indicated as blue bars. Nodes indicated by a black dot have a Bayesian posterior probability (BPP) of 1.00. Detailed results for all nodes are reported in Appendix S3 and node numbers on the tree correspond to those used there. Estimated age for the *Isoetes* crown group is 282 Myr (216–318 Myr). Our node nr. 11 corresponds with the crown group *Isoetes* as estimated by Wood et al. (2020), and the estimated age 119 Myr (59–170 Myr) is considerably older than the age of 23 Myr (6–47 Myr) obtained in their analyses.

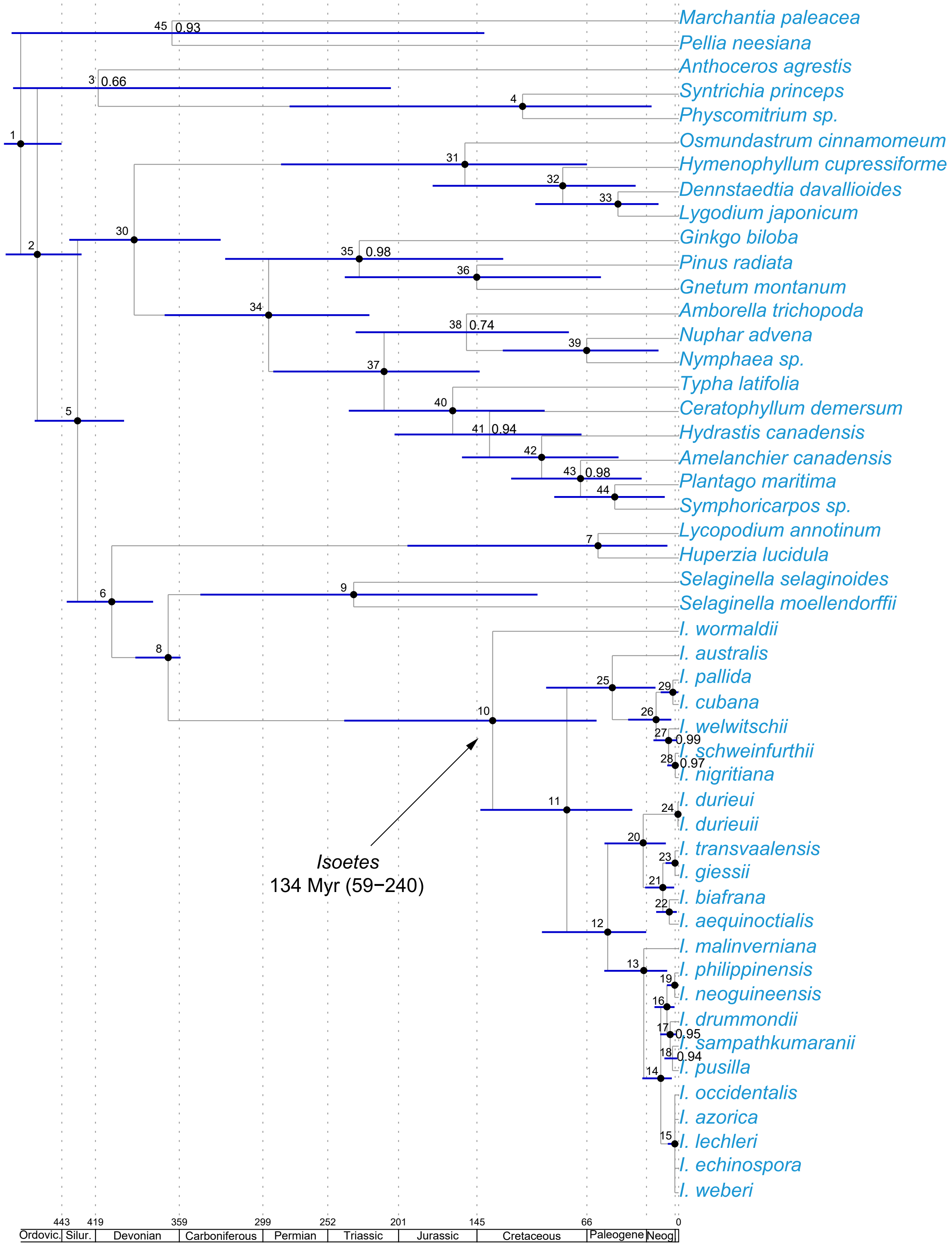

Figure S6 Chronogram showing estimated absolute ages resulting from the relaxed-clock analysis of nuclear rDNA gene data using the independent lognormal model (ILN) and a birth-death tree prior. Node heights are median ages. Confidence intervals (95% highest posterior density) of node ages are indicated as blue bars. Nodes indicated by a black dot are well supported and have a Bayesian posterior probability (BPP) of 0.95 or more. Detailed results for all nodes are reported in Appendix S4 and node numbers on the tree correspond to those used there. Estimated age for the *Isoetes* crown group is 134 Myr (59–240 Myr). Our node nr. 11 corresponds with the crown group *Isoetes* as estimated by Wood et al. (2020), and the estimated age 80 Myr (33–143 Myr) is somewhat older than the age of 54 Myr (28–85 Myr) obtained in their analyses.

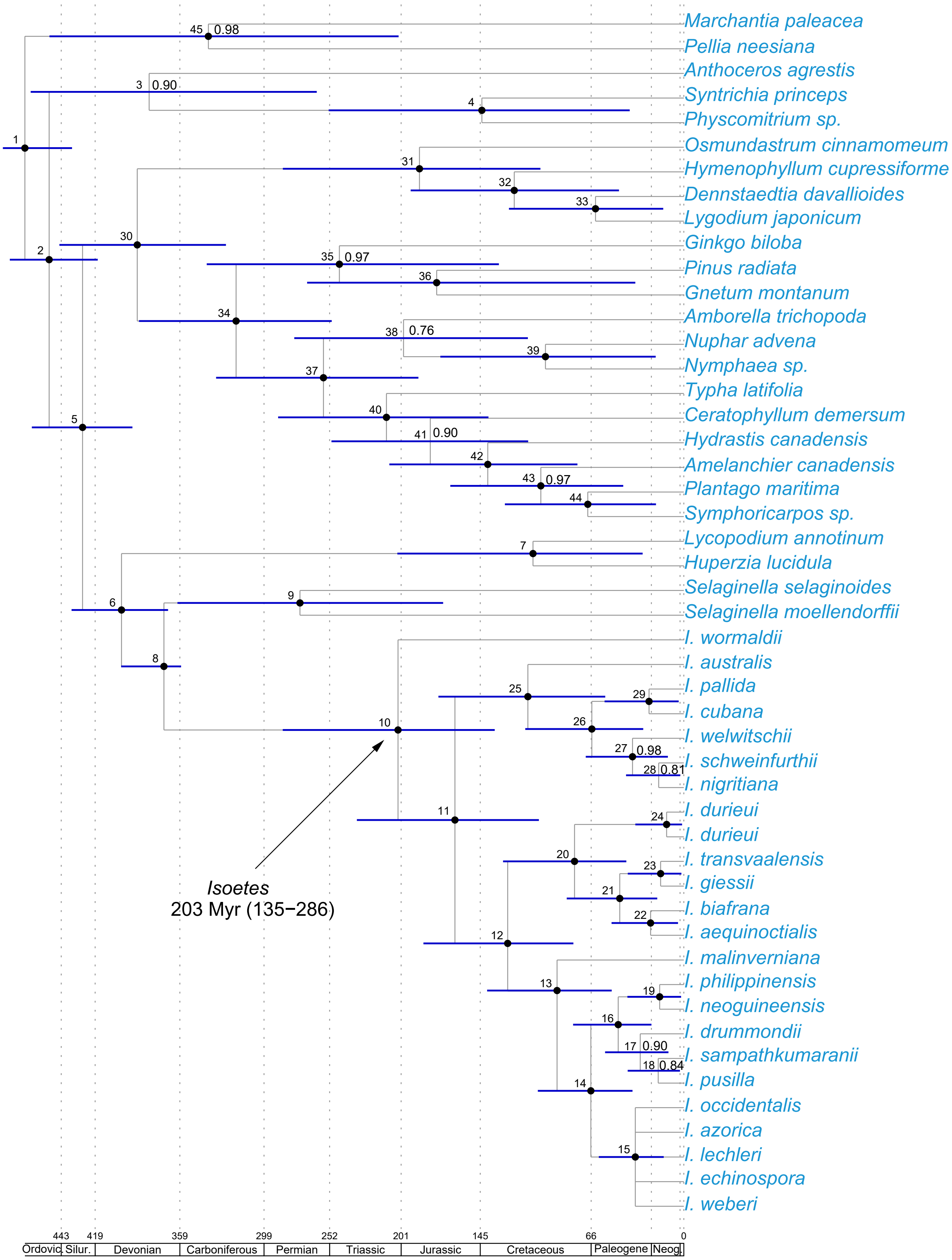

Figure S7 Chronogram showing estimated absolute ages resulting from the relaxed-clock analysis of nuclear rDNA gene data using the white noise model (WN) and a birth-death tree prior. Node heights are median ages. Confidence intervals (95% highest posterior density) of node ages are indicated as blue bars. Nodes indicated by a black dot are well supported and have a Bayesian posterior probability (BPP) of 0.95 or more. Detailed results for all nodes are reported in Appendix S4 and node numbers on the tree correspond to those used there. Estimated age for the *Isoetes* crown group is 203 Myr (135–286 Myr). Our node nr. 11 corresponds with the crown group *Isoetes* as estimated by Wood et al. (2020), and the estimated age 163 Myr (103–233 Myr) is considerably older than the age of 54 Myr (28–85 Myr) obtained in their analyses.

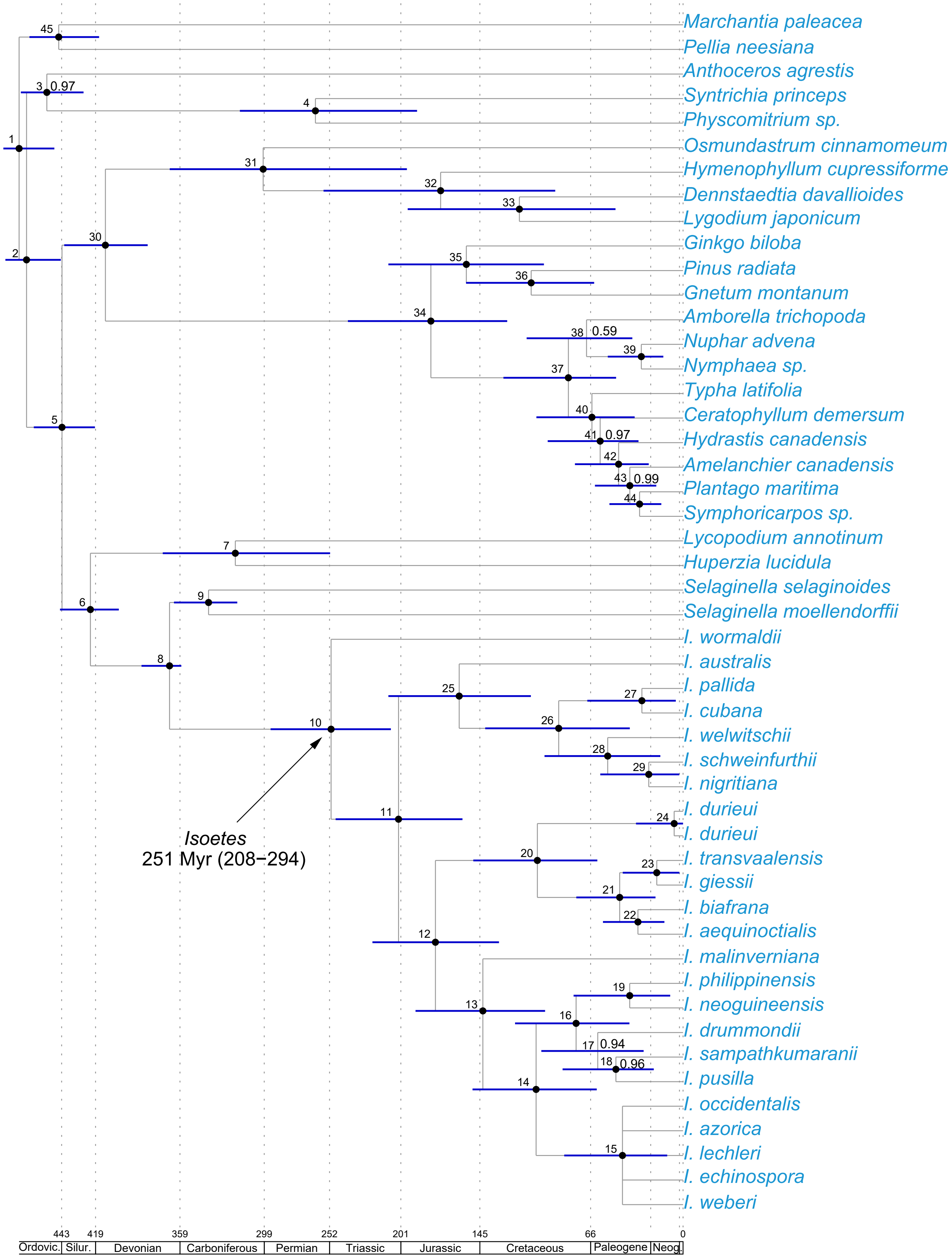

Figure S8 Chronogram showing estimated absolute ages resulting from the relaxed-clock analysis of nuclear rDNA gene data using the brownian motion model described by Thorne and Kishino (TK02) and a birth-death tree prior. Node heights are median ages. Confidence intervals (95% highest posterior density) of node ages are indicated as blue bars. Nodes indicated by a black dot are well supported and have a Bayesian posterior probability of 0.95 or more. Detailed results for all nodes are reported in Appendix S4 and node numbers on the tree correspond to those used there. Estimated age for the *Isoetes* crown group is 251 Myr (208–294 Myr). Our node nr. 11 corresponds with the crown group *Isoetes* as estimated by Wood et al. (2020), and the estimated age 203 Myr (157–248 Myr) is considerably older than the age of 54 Myr (28–85 Myr) obtained in their analyses.

References

Larsén, E., and C. Rydin. 2016. Disentangling the phylogeny of *Isoetes* (Isoetales), using nuclear and plastid data. *International Journal of Plant Sciences* 177: 157–174.
Larsén, E., N. Wikström, A. Khodabandeh, and C. Rydin. 2022. Phylogeny of Merlin's grass (Isoetaceae): revealing an "*Amborella* syndrome" and the importance of geographic distribution for understanding current and historical diversity. *BMC Ecology and Evolution* 22: 32.
Drummond, A. J., S. Y. W. Ho, M. J. Phillips, and A. Rambaut. 2006. Relaxed phylogenetics and dating with confidence. *PLoS Biology* 4: e88.
Wood, D., G. Besnard, D. J. Beerling, C. P. Osborne, and P.-A. Christin. 2020. Phylogenomics indicates the "living fossil" *Isoetes* diversified in the Cenozoic. *PLoS ONE* 15: e0227525.
LePage, T., D. Bryant, H. Philipp, and N. Lartillot. 2007. A general comparison of relaxed molecular clock models. *Molecular Biology and Evolution* 24: 2669–2680.
Thorne, J. L., and H. Kishino. 2002. Divergence time and evolutionary rate estimation with multilocus data. *Systematic Biology* 51: 689–702.
