# Supplementary material for: No phylogenomic support for a Cenozoic origin of the “living fossil” Isoetes
Source: Am J Bot. 2023 Jan 2;110(1):e16108. doi: 10.1002/ajb2.16108 (PMC10108322; doi:10.1002/ajb2.16108)
Supplement: Supplementary file 3 — Appendix S3. Detailed results from the individual divergence time analyses using complete plastid CDS data and alternative relaxed‐clock models (ILN, WN, and TK02; see text). [file AJB2-110-0-s004.pdf]

Appendix S3. Detailed results from the individual divergence time analyses using complete plastid CDS data and alternative relaxed clock models (ILN, WN, and TK02; see text). Node numbers correspond to those in Appendix S2 (Figures S3-S5). The Bayesian posterior probability (BPP), lineage median age, and the 95% HPD confidence intervals are reported for each node and analysis. Dashes (–) in the individual analyses indicate that the node was absent in the results.

		**ILN analyses**			**WN analyses**			**TK02 analyses**		
**Node**	**Clade/Taxon**	**BPP**	**Lineage age (Myr)**	**95% HPD of age (Myr)**	**BPP**	**Lineage age (Myr)**	**95% HPD of age (Myr)**	**BPP**	**Lineage age (Myr)**	**95% HPD of age (Myr)**
1	Land plants	1.00	478	457-485	1.00	477	456-485	1.00	483	475-485
2		1.00	468	444-484	1.00	464	440-483	1.00	478	467-485
3		1.00	141	44-280	1.00	159	68-279	1.00	342	291-392
4		1.00	440	413-465	1.00	445	412-472	1.00	429	418-440
5	Tracheophytes	1.00	415	391-440	1.00	424	396-460	1.00	406	397-416
6	Lycopsids	1.00	390	369-416	1.00	389	369-419	1.00	388	381-397
7	Lycopodiaceae	1.00	82	29-162	1.00	121	61-232	1.00	332	315-344
8		1.00	363	358-379	1.00	364	358-383	1.00	360	358-365
9	Selaginellaceae	1.00	227	140-281	1.00	224	131-288	1.00	211	168-250
10	Isoetaceae	1.00	54	27-99	1.00	150	91-211	1.00	282	216-318
11		1.00	21	13-36	1.00	101	67-139	1.00	119	59-170
12		1.00	15	10-25	1.00	77	48-110	1.00	100	44-154
13		1.00	11	6-17	1.00	60	37-87	1.00	81	26-119
14		1.00	6	3-9	1.00	47	29-74	1.00	44	14-72
15	*Isoetes* Clade E	1.00	2	1-4	1.00	31	17-50	1.00	11	3-24
16		0.86	2	1-4	0.55	24	10-42	0.74	11	3-23
17		1.00	1	1-2	1.00	17	5-31	1.00	6	2-13
18		1.00	1	0-2	1.00	9	1-20	1.00	4	1-9
19		1.00	0	0-1	1.00	11	1-23	1.00	2	0-6
20	*Isoetes* Clade D	1.00	3	2-6	1.00	33	17-54	1.00	32	9-56
21		0.88	3	1-5	0.87	24	9-41	0.88	31	9-54
22		1.00	2	1-4	1.00	13	2-26	1.00	22	6-40
23		1.00	1	0-2	1.00	13	2-28	1.00	9	2-21
24	*Isoetes* Clade B	1.00	7	2-13	1.00	42	19-69	1.00	34	10-71
25		1.00	1	0-2	1.00	22	8-41	1.00	4	1-12
26		1.00	0	0-0	0.85	6	1-16	0.88	0	0-0
27		1.00	0	0-0	1.00	7	1-17	1.00	0	0-1
28		1.00	0	0-0	1.00	6	0-18	1.00	0	0-1
29	*Isoetes* Clade A	1.00	13	7-22	1.00	63	32-98	1.00	75	18-121
30		1.00	3	1-8	1.00	32	13-60	1.00	28	4-68
31		1.00	0	0-0	1.00	11	3-24	1.00	1	0-8
32		0.63	0	0-0	–	–	–	0.70	0	0-3
33		1.00	1	0-3	1.00	14	2-31	1.00	11	1-32
34	Euphyllophytes	1.00	375	318-422	1.00	393	325-440	1.00	395	386-406
35	Ferns	1.00	265	177-349	1.00	269	196-330	1.00	269	194-332
36		1.00	185	111-287	1.00	202	159-266	1.00	167	95-212
37		1.00	133	67-230	1.00	157	100-219	1.00	135	77-184
38	Seed plants	1.00	265	180-323	1.00	285	211-340	1.00	330	306-330
39	Gymnosperms	1.00	202	121-275	1.00	220	146-289	1.00	302	278-336
40		1.00	138	64-209	1.00	162	82-236	1.00	231	199-266
41	Angiosperms	1.00	174	127-265	1.00	174	137-222	1.00	161	120-200
42		1.00	154	109-237	1.00	149	106-194	1.00	147	108-183
43		1.00	18	3-44	1.00	36	13-92	1.00	41	20-69
44		1.00	122	86-223	1.00	128	90-181	1.00	122	85-148
45		1.00	112	66-193	1.00	105	74-167	1.00	117	82-143
46		1.00	83	51-144	1.00	83	55-144	1.00	100	69-123
47		1.00	48	24-74	1.00	46	26-83	1.00	32	19-41
48		1.00	29	11-47	1.00	30	12-65	1.00	17	9-22
49		1.00	295	130-453	1.00	302	148-401	1.00	352	314-384
