# Supplementary material for: No phylogenomic support for a Cenozoic origin of the “living fossil” Isoetes
Source: Am J Bot. 2023 Jan 2;110(1):e16108. doi: 10.1002/ajb2.16108 (PMC10108322; doi:10.1002/ajb2.16108)
Supplement: Supplementary file 4 — Appendix S4. Detailed results from the individual divergence time analyses using nuclear ribosomal cistron data and alternative relaxed‐clock models (ILN, WN, and TK02; see text) [file AJB2-110-0-s002.pdf]

Appendix S4. Detailed results from the individual divergence time analyses using nuclear ribosomal cistron data and alternative relaxed-clock models (ILN, WN, and TK02; see text). Node numbers correspond to those shown in Appendix S2 (Figures S6-S8). The Bayesian posterior probability (BPP), lineage median age, and the 95% HPD confidence intervals are reported for each node and analysis. Dashes (–) in the individual analyses indicate that the node was absent in the results.

		**ILN analyses**			**WN analyses**			**TK02 analyses**		
**Node**	**Clade/Taxon**	**BPP**	**Lineage age (Myr)**	**95% HPD of age (Myr)**	**BPP**	**Lineage age (Myr)**	**95% HPD of age (Myr)**	**BPP**	**Lineage age (Myr)**	**95% HPD of age (Myr)**
1	Land plants	1.00	473	444-485	1.00	469	436-485	1.00	474	449-485
2		1.00	461	429-484	1.00	452	417-480	1.00	468	444-484
3		0.66	417	207-479	0.90	381	261-465	0.97	454	428-472
4		1.00	112	19-280	1.00	144	39-253	1.00	262	190-316
5	Tracheophytes	1.00	432	399-463	1.00	428	393-464	1.00	443	420-463
6	Lycopsids	1.00	407	378-440	1.00	400	367-436	1.00	423	403-445
7	Lycopodiaceae	1.00	58	8-195	1.00	107	29-204	1.00	319	252-371
8		1.00	367	358-391	1.00	370	358-401	1.00	366	358-386
9	Selaginellaceae	1.00	234	101-344	1.00	273	171-361	1.00	339	318-363
10	Isoetaceae	1.00	134	59-240	1.00	203	135-286	1.00	251	208-294
11		1.00	80	33-143	1.00	163	103-233	0.98	203	157-248
12		1.00	51	23-98	1.00	125	79-185	1.00	177	131-222
13		1.00	25	8-53	1.00	90	51-140	1.00	143	98-191
14		1.00	13	5-26	1.00	66	36-104	1.00	105	62-150
15	*Isoetes* Clade E	1.00	3	0-8	1.00	34	14-61	1.00	43	11-85
16	*Isoetes* Clade D	1.00	8	3-17	1.00	47	23-79	1.00	76	38-120
17		0.95	6	2-13	0.90	31	11-56	0.94	61	28-101
18		0.94	4	1-10	0.84	18	3-40	0.96	48	21-86
19		1.00	3	0-8	1.00	17	2-40	1.00	38	9-78
20	*Isoetes* Clade B	1.00	25	9-54	1.00	78	41-129	1.00	104	61-150
21		1.00	11	3-24	1.00	46	19-83	1.00	45	20-76
22		1.00	7	1-16	1.00	24	4-51	1.00	32	13-57
23		1.00	3	0-9	1.00	16	2-40	1.00	19	3-43
24		1.00	0	0-3	1.00	12	1-34	1.00	6	0-34
25	*Isoetes* Clade A	1.00	48	17-95	1.00	111	56-175	1.00	160	109-210
26		1.00	16	5-36	1.00	66	29-113	1.00	89	38-141
27		0.99	7	1-18	0.98	36	11-70	1.00	54	16-99
28		0.97	2	0-8	0.81	18	3-41	1.00	24	3-59
29		1.00	4	0-13	1.00	25	4-56	1.00	29	5-68
30	Euphyllophytes	1.00	391	329-438	1.00	389	326-445	1.00	412	382-442
31	Ferns	1.00	154	66-286	1.00	188	102-286	1.00	300	197-366
32		1.00	83	31-177	1.00	121	46-194	1.00	173	91-257
33		1.00	44	14-103	1.00	63	15-124	1.00	117	48-196
34	Seed plants	1.00	295	222-369	1.00	319	251-398	1.00	180	125-239
35	Gymnosperms	0.98	230	126-326	0.97	245	132-340	1.00	155	99-210
36		1.00	145	56-240	1.00	176	34-268	1.00	108	63-155
37	Angiosperms	1.00	212	143-291	1.00	257	189-333	1.00	82	48-128
38		0.74	153	79-232	0.76	200	111-277	0.59	69	36-112
39		1.00	66	14-126	1.00	98	20-173	1.00	30	14-54
40		1.00	162	96-237	1.00	212	139-289	1.00	65	35-105
41		0.94	136	70-204	0.90	181	111-251	0.97	59	32-97
42		1.00	98	43-156	1.00	140	76-210	1.00	46	24-77
43		0.98	71	27-120	0.97	102	43-166	0.99	38	19-63
44		1.00	46	10-89	1.00	68	20-127	1.00	31	16-53
45		0.93	364	140-480	0.98	339	203-452	1.00	445	417-466
